# Grape Ripening Is Regulated by Deficit Irrigation/Elevated Temperatures According to Cluster Position in the Canopy

**DOI:** 10.3389/fpls.2016.01640

**Published:** 2016-11-15

**Authors:** Olfa Zarrouk, Cecilia Brunetti, Ricardo Egipto, Carla Pinheiro, Tânia Genebra, Antonella Gori, Carlos M. Lopes, Massimiliano Tattini, M. Manuela Chaves

**Affiliations:** ^1^Instituto de Tecnologia Química e Biológica, Universidade NOVA de LisboaOeiras, Portugal; ^2^Trees and Timber Institute, The National Research Council of ItalyFlorence, Italy; ^3^Department of Plant, Soil and Environmental Sciences, University of FlorenceFlorence, Italy; ^4^Linking Landscape, Environment, Agriculture and Food (LEAF), Instituto Superior de Agronomia, Universidade de LisboaLisboa, Portugal; ^5^Faculdade de Ciências e Tecnologia, Universidade NOVA de LisboaCaparica, Portugal; ^6^Department of Biology, Agriculture and Food Sciences, Institute for Sustainable Plant Protection, The National Research Council of ItalyFlorence, Italy

**Keywords:** ABA metabolism, anthocyanins, flavonols, heat stress *Vitis vinifera*, water stress

## Abstract

The impact of water deficit on berry quality has been extensively investigated during the last decades. Nonetheless, there is a scarcity of knowledge on the performance of varieties exposed to a combination of high temperatures/water stress during the growing season and under vineyard conditions. The objective of this research was to investigate the effects of two irrigation regimes, sustained deficit irrigation (SDI, 30% ET_c_) and regulated deficit irrigation (RDI, 15% ET_c_) and of two cluster positions within the canopy (east- and west-exposed sides) on berry ripening in red Aragonez (Tempranillo) grapevines. The study was undertaken for two successive years in a commercial vineyard in South Portugal, monitoring the following parameters: pre-dawn leaf water potential, berry temperature, sugars, polyphenols, abscisic acid (ABA) and related metabolites. Additionally, expression patterns for different transcripts encoding for enzymes responsible for anthocyanin and ABA biosynthesis (*VviUFGT, VvNCED1, Vv*β*G1, VviHyd1, VviHyd2*) were analyzed. In both years anthocyanin concentration was lower in RDI at the west side (RDIW- the hottest one) from *véraison* onwards, suggesting that the most severe water stress conditions exacerbated the negative impact of high temperature on anthocyanin. The down-regulation of *VviUFGT* expression revealed a repression of the anthocyanin synthesis in berries of RDIW, at early stages of berry ripening. At full-maturation, anthocyanin degradation products were detected, being highest at RDIW. This suggests that the negative impact of water stress and high temperature on anthocyanins results from the repression of biosynthesis at the onset of ripening and from degradation at later stages. On the other hand, berries grown under SDI displayed a higher content in phenolics than those under RDI, pointing out for the attenuation of the negative temperature effects under SDI. Irrigation regime and berry position had small effect on free-ABA concentration. However, ABA catabolism/conjugation process and ABA biosynthetic pathway were affected by water and heat stresses. This indicates the role of ABA-GE and catabolites in berry ABA homeostasis under abiotic stresses. Principal component analysis (PCA) showed that the strongest influence in berry ripening is the deficit irrigation regime, while temperature is an important variable determining the improvement or impairment of berry quality by the deficit irrigation regime. In summary, this work shows the interaction between irrigation regime and high temperature on the control of berry ripening.

## Introduction

The effect of water stress on grape berry ripening and quality has been extensively investigated during the last decades (for reviews, see Chaves et al., 2010; Kuhn et al., 2013). Overall, berry quality benefits from mild to moderate water deficit (Chaves et al., 2010) and the reduction in berry development was early proposed as mostly responsible for the improvement of berry quality by water deficits (Matthews and Anderson, 1988). However, more recently water deficit was shown to profoundly alter berry secondary metabolism, particularly of flavonoids, thus greatly regulating the ripening process (development and composition) (Castellarin et al., 2007a,b). The regulation of genes and proteins of the various metabolic pathways is either the consequence of a direct effect of water shortage and/or indirect via the changing of the light environment around grape clusters due to the impairment of vine vegetative growth.

The matter is far from being conclusively addressed, two major causes can be highlighted: the experimental set-ups adopted in the majority of experiments, namely the water stress timing and conditions; the diversity of varieties (Zarrouk et al., 2016). It was shown that pre-*véraison* water stress promotes the accumulation of anthocyanins, as a consequence of both reduced berry development and enhanced expression of flavonoid genes, thus leading (Basile et al., 2011; Intrigliolo et al., 2012; Romero et al., 2013) to earlier grape ripening (Castellarin et al., 2007a,b). In contrast, water stress imposed at post-*véraison* just increased the proportion of seeds and skin relative to whole-berry fresh mass, without significant effects on secondary metabolism (Roby and Matthews, 2004).

On the other hand, several reports (Deluc et al., 2009; Girona et al., 2009; Niculcea et al., 2014) have shown that the effect of water deficit on the biosynthesis of phenolics and berry growth is strongly cultivar dependent. The differential increase of anthocyanin compounds in berries under pre- or post-*véraison* deficit irrigation was primarily attributed to large differences in abscisic acid (ABA) sensitivity displayed by different varieties (Ferrandino and Lovisolo, 2013). Grape varieties may have either isohydric or anisohydric strategy to cope with water stress, as a consequence of large differences in the ability to regulate water losses through chemical (i.e., ABA) signaling (Deluc et al., 2009; Lovisolo et al., 2010; Balint and Reynolds, 2013; Niculcea et al., 2014). In addition, ABA was reported to strongly control key events of grape ripening through regulation of primary (sugars) and secondary metabolite biosynthesis (e.g., anthocyanins) (Davies et al., 1997; Peppi and Fidelibus, 2008). ABA levels peak around *véraison* stage (Owen et al., 2009; Wheeler et al., 2009), following the up-regulation of gene transcription and proteins expression related to ABA biosynthesis (Deluc et al., 2007; Giribaldi et al., 2007). These findings support the idea that ripening of non-climacteric grapes is, to a certain extent, ABA-regulated (Davies et al., 1997; Jia et al., 2011; Niculcea et al., 2014). Nonetheless, the expression of 9-cis-epoxycarotenoid dioxygenase (NCED), the enzyme involved in the first committed step of ABA (Qin and Zeevaart, 2002) is not always correlated with the concentration of free-ABA in berries (Deluc et al., 2009; Wheeler et al., 2009). This suggests that products of ABA catabolism/conjugation (Nambara and Marion-Poll, 2003) may be also involved in berry ripening. Free-ABA, the active form of this hormone, may be oxidized by ABA 8′-hydroxylase to phaseic (PA) and dihydrophaseic acids (DPA) or conjugated with glucose to form ABA-glucosylester (ABA-GE), through the action of ABA glucosyltransferase (ABA-GTase). ABA-GE plays an important role in the regulation of ABA content since it releases free-ABA through β-D-glucosidase and it has been proposed to be involved in long-distance transport of ABA.

Several reports showed the impact of water shortage on the hormonal balance of grape berry during ripening (Deluc et al., 2009; Niculcea et al., 2013) and on the ABA catabolites concentrations (Balint and Reynolds, 2013). Zarrouk et al. (2012) showed that both free ABA and ABA-GE are involved in the enhancement of ripening process in stressed berries.

In this context, Coombe (1989) attributes the promotion of sugar accumulation in water stressed berries to a direct effect of ABA signaling on fruit ripening. More recently, Niculcea et al. (2014) observed that the imposition of both types of deficit irrigation (pre- and post-*véraison*), altered the pattern of ABA accumulation, and relates the improvement of sugar concentration, phenolic substances and anthocyanins to the persistence of ABA production over time in post-*véraison*-stressed berries. Nonetheless, the enhancement of berry ABA content by water stress is not always accompanied by an increase in sugars or in secondary metabolites. In our previous investigation (Zarrouk et al., 2012), a negative effect of moderate deficit irrigation on grape berry anthocyanin concentration was observed, despite the increase in ABA concentration at *véraison*, suggesting a decoupling on the ripening process parameters due to other external factor than water deficit. Climate condition, namely high temperature along the growing season is considered a putative constraint to the implementation and success of the deficit irrigation regime (Shellie, 2011). Also, the interaction between elevated temperature and water deficit is considered the main cause of variability in field experiments results (Bonada et al., 2015) namely in what concerns the ripening of wine grapes (Real et al., 2015).

In this context, Fernandes de Oliveira and Nieddu (2013) showed that high temperature at mid-ripening coupled with moderate deficit irrigation (25% of Etc) reduced total anthocyanin content, possibly by degrading these compounds or/and inhibiting their biosynthesis. Bonada et al. (2015) related an advance of the onset of berry net water loss by elevated temperature under water deficit, which hastened berry ripening and altered the balance of sensory traits.

The effect of temperature on berry composition has been studied extensively and the negative impact of high temperature on anthocyanin content was reported (Spayd et al., 2002; Mori et al., 2007; Sadras and Moran, 2012; Bonada and Sadras, 2014; Bonada et al., 2015). Nonetheless, to date, there appears to be a scarcity of knowledge of the performance of different varieties exposed to a combination of high temperatures and water stress during the growing seasons and under vineyard conditions (Greer and Weedon, 2013).

Portugal is the 11th highest wine-producing country worldwide (OIV, 2016), with strong influence of wine industry on the economic stability and development of the country (Fraga et al., 2016). Recent climate studies in Portugal highlight an increase in the temperature during grape growing season (Fraga et al., 2012), with increase in minimum temperature during the ripening period (Malheiro et al., 2010; Fraga et al., 2012) and an acute dryness in summer (Santos et al., 2012). This compromise the optimal climatic conditions to grow most current varieties particularly in southern Portugal (Real et al., 2015), with likely negative impact in wine characteristics. Irrigation is being used to maintain winegrape yield and overcome the negative impact of water stress incidence during the grapevine reproductive season, but the effects of different kinds of irrigation systems on berry and wine quality are still largely unknown.

In this investigation we analyzed the combined effect of water and heat stress in grape berry ripening process. Our experiments with Aragonez variety (syn. Tempranillo) over two consecutive seasons aimed at the revelation of the existence of differences in the accumulation and the biosynthesis of different parameters that define ripening in relation to the cluster position within the canopy and the irrigation regime applied. For this, the effect of two different irrigation regimes on grape berry skin during development was examined. The study focused on sugar accumulation in the berry, anthocyanin and flavonol accumulation in skin, endogenous ABA and ABA metabolites, as well as expression patterns for different transcripts encoding for enzymes responsible for anthocyanin and ABA biosynthesis (*VviUFGT, VvNCED1, Vv*β*G1, VviHyd1, VviHyd2*).

## Materials and methods

### Vineyard site, experimental design, and berry temperature measurements

Experiments were conducted over 2013-2014 in a commercial vineyard (Herdade do Esporão), located at Reguengos de Monsaraz, Alentejo winegrowing region, Southern Portugal (lat. 38° 23′ 55.00″ N; long. 7° 32′ 46.00″ W). The climate is of Mediterranean type, with hot and dry summers and mild rainy winters. Soil texture is a sandy-loam to silty-clay-loam, with a pH of 7.0–7.6, a low content in organic matter (10.5 g kg^−1^) and high P_2_O_5_ and K_2_O values (110 and 173 mg kg^−1^, respectively). The 11-year-old grapevines of the red variety Aragonez (syn. Tempranillo) were grafted on 1103 Paulsen rootstock. Vines were spaced 1.5 m within and 3.0 m between rows, on a north-south orientation, trained on a vertical shoot positioning and spur-pruned on a bilateral Royat Cordon system. All vines were uniformly pruned with 15–16 nodes per vine. The experimental layout was a randomized complete block design with two treatments and four replications per treatment. The elemental plot comprised three adjacent rows (two buffer rows and a central one for data collection) of 10 vines each. Climate data (air temperature and precipitation) were obtained from an automatic meteorological station located in the experimental orchard, near the vineyard (at ~900 m).

Two irrigation treatments were applied: Sustained Deficit Irrigation (SDI), usually applied by winegrower, and Regulated Deficit Irrigation (RDI). The drip line was positioned along the row close to the vine trunks and consisted of pressure compensating 2.2 L/h drip emitters with 1.0 m spacing. Irrigation started on June, 14th and was ended at September, 6th in 2013; in 2014 it started on June, 12th and stopped on August, 23th. Watering was applied according to the crop evapotranspiration (ET_c_) and soil water content. ET_c_ was estimated from the reference evapotranspiration (ET_0_) using the crop coefficients proposed by Allen et al. (1998). During irrigation, the average fraction of ET_c_ applied was *ca* 0.3 in SDI and 0.15 in RDI. Water was applied 1–2 times a week. The total amount of water supplied until commercial harvest to SDI plants was 111 and 57 mm in 2013 and 2014 respectively (~30% ET_c_), while the supply on RDI was 53 mm in 2013 and 38 mm in 2014 (~15% ET_c_) (Supplementary Table 1). Standard cultural practices in the region were applied to all treatments. To characterize the vine water status, pre-dawn leaf water potential (Ψ_pd_) was measured before each sampling date. Measurements were carried out on an adult leaf from eight replicate plants from each treatment, using a Scholander pressure chamber (Model 1000; PMS instrument Co., Corvallis, OR, USA). In order to estimate accumulated water stress, pre-dawn water stress integral (SΨ_pd_) was determined for each phenological stage accordingly to Myers (1988), using a −0.2 MPa maximum threshold. Berry temperature was monitored continuously (at 30-min-intervals) using a dual channel temperature data logger to which a two-junction, fine wires copper-constantan thermocouples were attached. These probes were positioned on berry surface (avoiding direct sun exposure) of a sample of clusters located in different canopy positions (exposed and internal; facing east and west) of 2 vines per treatment.

In order to quantify the incidence of berry temperature on anthocyanin accumulation at each side of the canopy (east and west), the Rustioni et al. (2011) approach was used. Berry temperature was averaged each hour and then converted into normal heat hours (NHH) and cumulated per phenological period. NHH computing procedure was done as described by Rustioni et al. (2011), based on a function that varies from 0 to 1 and gives 0 for temperatures outside minimum and maximum cardinals (T_min_ = 10°C and T_max_ = 35°C) and 1 for temperatures at optimum (T_opt_ = 25°C).

### Grape berry analysis

Berries were collected at different developmental stages during summer (from June to August). Four stages were considered: end of pea size (PS, 5–6 weeks after anthesis), *véraison* (V, 9–10 weeks after anthesis), mid-ripening (MR, 12 weeks after anthesis) and full maturation (FM, 14 weeks after anthesis). During 2013, only three stages were sampled (pea size, *véraison* and full maturation) as a consequence of unusual early berry maturation and an acceleration of sugar accumulation and acid breakdown. At each sampling date a representative sample of 50 bunches per treatment from 10 vines per replicate (30-40 plants per treatment) was randomly collected from the experimental vineyard. Bunches from each east side and west side of the vine (East side and West side) were collected separately. Four sample groups were generated: SDI east (SDIE), SDI west (SDIW), RDI east (RDIE), and RDI west (RDIW). A sub-sample of 20 bunches was collected and stored at 4°C. Three independent replicates of 50 berries each were weighed to determine berry weight and diameter, and the juice was extracted to determine total soluble solids (TSS, °Brix) and titratable acidity (TA, g tartaric acid L^−1^). The concentration of TSS was measured using a manual refractometer (ITREF 32, Instrutemp). The TA was assessed according to Office International de la Vigne et du Vin (OIV, 1990) procedure. The second sub-sample of 30 bunches was immediately frozen in liquid nitrogen from which three to four independent pools of 30-40 frozen berries were carefully selected, peeled and the seeds removed. Skins were removed, weighted and ground in liquid nitrogen to a fine powder and stored at −80°C until successive analysis.

### ABA and related metabolites analysis

ABA, ABA-GE, PA, and DPA concentrations in berry skin were simultaneously analyzed by HPLC MS/MS. Before starting the extraction procedure, 40 ng of each deuterium-labeled internal standards were added (ABA-d6, ABA-GE-d5, PA-d3, DPA-d3, all from National Research Council of Canada, Saskatoon, SK, Canada) per 300 mg of freeze dried skin powder. The tissue was extracted with 3 mL of CH_3_OH:H_2_O (50:50 adjusted to pH 2.5 with HCOOH) for 30 min at 4°C. The supernatant was partitioned three times with 3 mL of n-exane. The methanolic fraction was purified using Sep-Pak C18 cartridges (Waters, Massachusetts, USA), and the eluate was dried under nitrogen and rinsed with 250 μL of CH_3_OH:H_2_O acidified at pH 2.5. Identification and quantification of free-ABA, ABA-GE, PA and DPA was performed through the injection of 3 μL of sample solution in a LC-ESI-MS/MS equipment, consisting of a LC-MS-8030 triple quadrupole mass spectrometer operating in negative ion mode equipped with electrospray ionization source (ESI) and coupled with a Nexera HPLC system (all from Shimadzu, Kyoto, Japan). Compounds were separated in an Agilent Poroshell C18 column (3.0 × 100 mm, 2.7 μm) eluted with a linear gradient solvent, at a flow rate of 0.3 mL min-1, from 95% H_2_O (with the addition of 0.1% of HCOOH, solvent A) to 100% CH_3_CN/MeOH (50/50, with the addition of 0.1% of HCOOH, solvent B) over a 30-min run. Quantification of free-ABA, ABA-GE, PA, and DPA was conducted in multiple reaction monitoring (MRM) with the corresponding transitions for each analyte (ABA: 263/153; d6-ABA: 269/159; ABA-GE: 425/263; d5-ABA-GE: 430/268; PA: 279/139; d3-PA: 282/142; DPA: 281/171; d3-DPA: 284/174).

### Phenylpropanoids analysis

Freeze dried skins (300–350 mg) were ground in a mortar under liquid nitrogen and the obtained powder was extracted with 70% of aqueous ethanol acidified to pH 2 by HCOOH (3 × 5 mL) and sonicated for 30 min. The supernatant was partitioned with 3 × 5 mL of n-Hexane, reduced to dryness under vacuum and rinsed with CH_3_OH/H_2_O (50/50, pH 2). Aliquots of 5 μL were injected into Perkin Elmer Flexar HPLC equipped with a quaternary 200Q/410 pump and a LC 200 photodiode array detector (Perkin Elmer, Bradford, CT, USA). Metabolites were separated in a 4.6 × 250 mm Hypersil SB-C_18_ column (5 μm) (Agilent Technologies, Milan, Italy), operating at 30°C and eluted at a flow rate of 1 mL min^−1^. Anthocyanins were separated using a gradient solvent system consisting of H_2_O (plus 5% HCOOH) (A), CH_3_OH (plus 5% HCOOH) (B), CH_3_CN (plus 5% HCOOH (C), during a 47 min run: 0–2 min 80% A, 15% B, 5% C; 2–17 min to 70% A, 20% B, 10% C; 17–32 min to 55% A, 30% B, 15% C; 32–37 min 55% A, 30% B, 15% C; 37–42 min to 20% A, 40% B, 40% C; 42–47 min 20% A, 40% B, 40% C. Flavonoids and hydroxycinnamic acids were separated using a linear gradient solvent system consisting of solvent A (90% H_2_O with the addition of 0.1% formic acid, 10% CH_3_CN) and solvent B (90% CH_3_CN and 10% of H_2_O with the addition of 0.1% formic acid). The chromatographic run lasted 45 min starting from 100% solvent A and arriving to 100% solvent B. Individual metabolites were identified on the basis of their retention times, UV-spectral characteristics and mass-spectrometric data. HPLC-MS-MS analysis was performed with a LC-MS-8030 triple quadrupole mass spectrometer operating in the electrospray ionization (ESI) mode and a Nexera HPLC system (all from Shimadzu, Kyoto, Japan). The mass spectrometer operated in negative ion scan mode for hydroxycinnamic derivatives and flavonoids detection, and in positive ion scan mode for anthocyanin detection. Product ion spectra were obtained using Argon as collision gas at a pressure of 230 kPa. Quantification of anthocyanins was performed at 530 nm using calibration curves of cyanidin 3-*O*-glucoside chloride, delphinidin 3-*O*-glucoside chloride, petunidin 3-*O*-glucoside chloride, malvidin 3-*O*-glucoside chloride (Extrasynthese). Quantification of flavonoids and hydroxycinnamic acid derivatives was performed at 350 and 330 nm respectively using the calibration curve of quercetin 3-*O*-glucoside, rutin, myricetin 3-*O*-glucoside, *trans*-caftaric acid and *trans*-coutaric acid; gallic acid, protocatechuic acid, vanillic acid and syringic acidwere quantified at 280 nm.

### Analysis of degradation products of anthocyanins

Degradation products of anthocyanins were quantified on the samples extracted for phenylpropanoids analysis following the protocol reported in Seeram et al. (2001) and Sadilova et al. (2007) with some modifications. Aliquots of 20 μL were injected into Perkin Elmer Flexar HPLC reported above and analyzed on a 4.6 × 250 mm Hypersil SB-C_18_ column (5 μm) (Agilent Technologies, Milan, Italy), operating at 30°C and eluted at a flow rate of 1 mL min^−1^. Degradation products of anthocyanins were detected at 235 nm and quantified using authentic standards of protocatechuic acid and phloroglucinaldehyde (Sigma Aldrich, Milan, Italy).

### RNA extraction and cDNA synthesis

Total RNA extractions were performed in 1.5 mL tube, using the method of Reid et al. (2006). Briefly, skin tissue of 20 berries was ground to a fine powder in liquid nitrogen using a mortar and pestle. The extraction buffer, pre-warmed (65°C) (300 mM Tris HCl pH 8.0, 25 mM EDTA, 2 M NaCl, 2% (w/v) CTAB, 2% (w/v) PVPP, 0.05% (w/v) spermidine trihydrochloride and 2% (v/v) β-mercaptoethanol just prior use) was added to powder and shaken vigorously. Tubes were subsequently incubated at 65°C with shaking for 10 min. Mixtures were extracted twice with equal volumes of chloroform:isoamyl alcohol (24:1) and centrifuged at 16,100 g for 10 min at 4°C. To the supernatant add 100 μl 3 M NaOAc (pH 5.2) and 600 μl isopropanol were added, mixed, and stored at −80 °C for 25 min. Nucleic acid pellets were collected by centrifugation at 16,100 g for 30 min at 4°C. The pellet was dissolved in 250 μL TE (pH 7.5) and adds 94 μL of 8 M LiCl and stored at 4°C overnight. RNA was pelleted by centrifugation at 16,100 g for 30 min at 4°C, then washed with 1 μl of ice cold 70% ethanol, air dried, and dissolved in RNase-free water. Total RNA was purified using an RNeasy® Mini kit (Qiagen) with the addition of an on-column DNAse I digestion (RNase-Free DNase Set; Qiagen). RNA concentration was determined before and after DNase I digestion using a Nanodrop ND-1000 spectrophotometer (Nanodrop Technologies) in 260/280 nm ratio. RNA integrity was evaluated by 1% (w/v) agarose gel electrophoresis. First-strand cDNA was synthesized using the Omniscript® Reverse Transcription kit (Qiagen) according to the manufacture's instructions. The cDNA was prepared from 1000 ng of total RNA and synthesized at 37°C for 60 min and the cDNA stored at −80°C.

### Quantitative real-time PCR analysis

Quantitative real-time PCR was performed in the iQ5 2.0 Standard Edition (Bio-Rad), sequence detection system in a 96-well reaction plate. Each reaction (20 μl) contained 250 nM of each primer, 5 μL of 1/50 diluted cDNA, and 10 μl of Power SYBR Green Master Mix (Bio-Rad). Thermal cycling conditions were 95°C for 10 min followed by 95°C for 10 s, 60°C for 10 s, and 72°C for 10 s for 40 cycles. Dissociation curves for each amplicon were then analyzed to verify the specificity of each amplification reaction; the dissociation curve was obtained by heating the amplicon from 55 to 95°C. Each PCR was run in triplicate within the same plate, and the cycle threshold (Ct) values obtained from the technical replicates were averaged. Gene transcripts were quantified by comparing the Ct of the target gene with that of actin (Reid et al., 2006). Gene expression was expressed as mean and standard error calculated from the three biological replicates. Primer pairs for *VviUFGT* were retrieved from Jeong et al. (2004), *VviNCED1* and *Vvi*β*G1* from Sun et al. (2010), *VviHyd1 and VviHyd2* from Speirs et al. (2013).

### Data analysis

Measurements were conducted of four biological replicates. Data of each season were subjected to analysis of variance (ANOVA) using SPSS 12.0 (SPSS Inc., Chicago, USA). Means were separated by Duncan's multiple range test (*p* ≤ 0.05). Principal component analysis (PCA) was also performed on all measured skin parameters, using the R software (version 3.2.5, R Development Core Team 2011) and the ade4 package (Culhane et al., 2002; Chessel et al., 2004; Thioulouse and Dray, 2009).

## Results

### Vine water status and cluster zone microclimate

Water applied in RDI treatment from pea-size berry to full maturation was similar in both years, while SDI treated-plants in 2014 received ~65% of water than in 2013 (Supplementary Table 1). The cumulative pre-dawn water stress (SΨ_pd_) was higher in 2014. In RDI and SDI treatments, accumulated stress from pea-size berry to *véraison* was 2–13 times higher in 2014 than in 2013, respectively (Table 1). The accumulated stress from *véraison* to full maturation was similar in both years in RDI treatment, while accumulated stress in SDI treatment in 2014 was 1.5 times higher than in 2013 (Table 1). Berry temperature (T_berry_) on west-exposed clusters was always higher than in east-exposed berries, irrespective of the phenological stage, irrigation treatment and year (Table 2; Supplementary Figure 1). Air temperature (T_air_) measured from *véraison* to full maturity was, on average, 2°C higher in 2013 than in 2014 (Supplementary Table 2). In the same period, maximum T_air_ was 0.4°C higher in 2014, while minimum temperature in 2013 was 1.0 °C higher than in 2014. In both years, the T_air_ never went below 10 °C. In 2013, an accumulated period of 121 h with T_air_ above 35°C was observed, whereas in 2014 only 83 h had T_air_ above 35°C. Though accumulated hours with T_air_ > 35°C in 2013 was higher, the duration of T_berry_ > 35°C observed in 2013 was lower than in 2014, irrespective of irrigation and cluster exposure.

**Table 1:** **Pre-dawn water stress integral (SΨpd), according to Myers (1988) in sustained deficit irrigation (SDI) and regulated deficit irrigation (RDI) treatments during the periods pea size -***véraison*** (PS-V), ***véraison***-mid-ripening (V-MR), mid-ripening-full maturation (MR-FM) and ***véraison***-full maturation (V-FM) in 2013 and 2014 growing seasons**.

		**S**Ψ_**pd**_**(MPa.day)**	
		**RDI**	**SDI**
2013	PS—V (DOY 191–210)	3.52	0.38
	V—FM (DOY 211–230)	6.43	3.75
2014	PS—V (DOY 167–195)	7.12	5.05
	V—MR (DOY 196–210)	7.95	6.05
	MR—FM (DOY 211–223)	5.87	4.79

*DOY: day of the year*.

**Table 2:** Maximum (T_**berry**_ max), Minimum (T_**berry**_ min) and Average (T_**berry**_ avg) berry temperature and Number of hours with temperature below 10°C (T_**berry**_ <10°C)^*^ and above 35°C (T_**berry**_ > 35°C) of east- and west-exposed clusters in sustained deficit irrigation (SDI) and regulated deficit irrigation (RDI) treatments during ***véraison*** (PS-V), mid-Ripening (V-MR), full maturation (MR-FM) and from ***véraison*** to full maturation (V-FM) period in 2013 and 2014 growing seasons.

**Year**	**Phenology**	**SDIE**				**SDIW**				**RDIE**				**RDIW**			
		**T_berry_**	**T_berry_**	**T_berry_**	**T_berry_ >**	**T_berry_**	**T_berry_**	**T_berry_**	**T_berry_ >**	**T_berry_**	**T_berry_**	**T_berry_**	**T_berry_ >**	**T_berry_**	**T_berry_**	**T_berry_**	**T_berry_ >**
		**max**	**min**	**avg**	**35°C**	**max**	**min**	**avg**	**35°C**	**max**	**min**	**avg**	**35°C**	**max**	**min**	**avg**	**35°C**
		**(°C)**	**(°C)**	**(°C)**	**(Hours)**	**(°C)**	**(°C)**	**(°C)**	**(Hours)**	**(°C)**	**(°C)**	**(°C)**	**(Hours)**	**(°C)**	**(°C)**	**(°C)**	**(Hours)**
2013	PS-V	39.2	10.6	24.3	22	47.2	10.3	24.5	45	37	10.6	24.1	10	46.2	10.4	24.9	48
	V-FM	42.5	12.6	26.4	88	48.2	12.4	26.7	112	40	12.7	26	73	47.8	12.3	26.8	106
	PS-FM	42.5	10.6	25.5	110	48.2	10.3	25.8	157	40	10.6	25.2	83	47.8	10.4	26	154
2014	PS-V	40.6	9.8	22.3	48	45.5	10.7	22.5	51	40.8	10.9	22.3	44	43.3	11	22.1	44
	V-MR	42.8	12.2	25.4	70	47.7	12.6	25.7	73	40	13.1	25.1	49	46.5	13.1	25.4	69
	MR-FM	38.4	12.2	25.3	35	46.9	12.2	24.9	48	36.6	12.9	25.1	20	42.8	12.8	25.3	56
	PS-FM	42.8	9.8	23.8	153	47.1	10.7	23.9	172	40.8	10.9	23.7	113	46.5	11	23.7	169

* *Except for SDIE regime in 2014 the number of hours with temperature below 10°C (T_berry_ < 10°C) were null. For SDIE regime in 2014 it was 1 at PS-V*.

Slight differences were observed between irrigation treatments in T_berry_ from clusters from the same canopy side in both years (Table 2). While the west-exposed clusters displayed maximum T_berry_ higher than the east exposed clusters, the amplitude was larger in the RDI treatment (T_berry_ 5–8°C and 2.5–9°C higher in SDI and RDI, respectively). Regarding for the number of hours with T_berry_ > 35°C, the berries of the RDI treatment were shown to accumulate more hours with T_berry_> 35°C than the berries of the SDI treatment. Although in 2014, the accumulated hours of berry temperature above 35°C was lower than the observed in 2013 (Table 2).

### Berry composition

**Figure 1:**
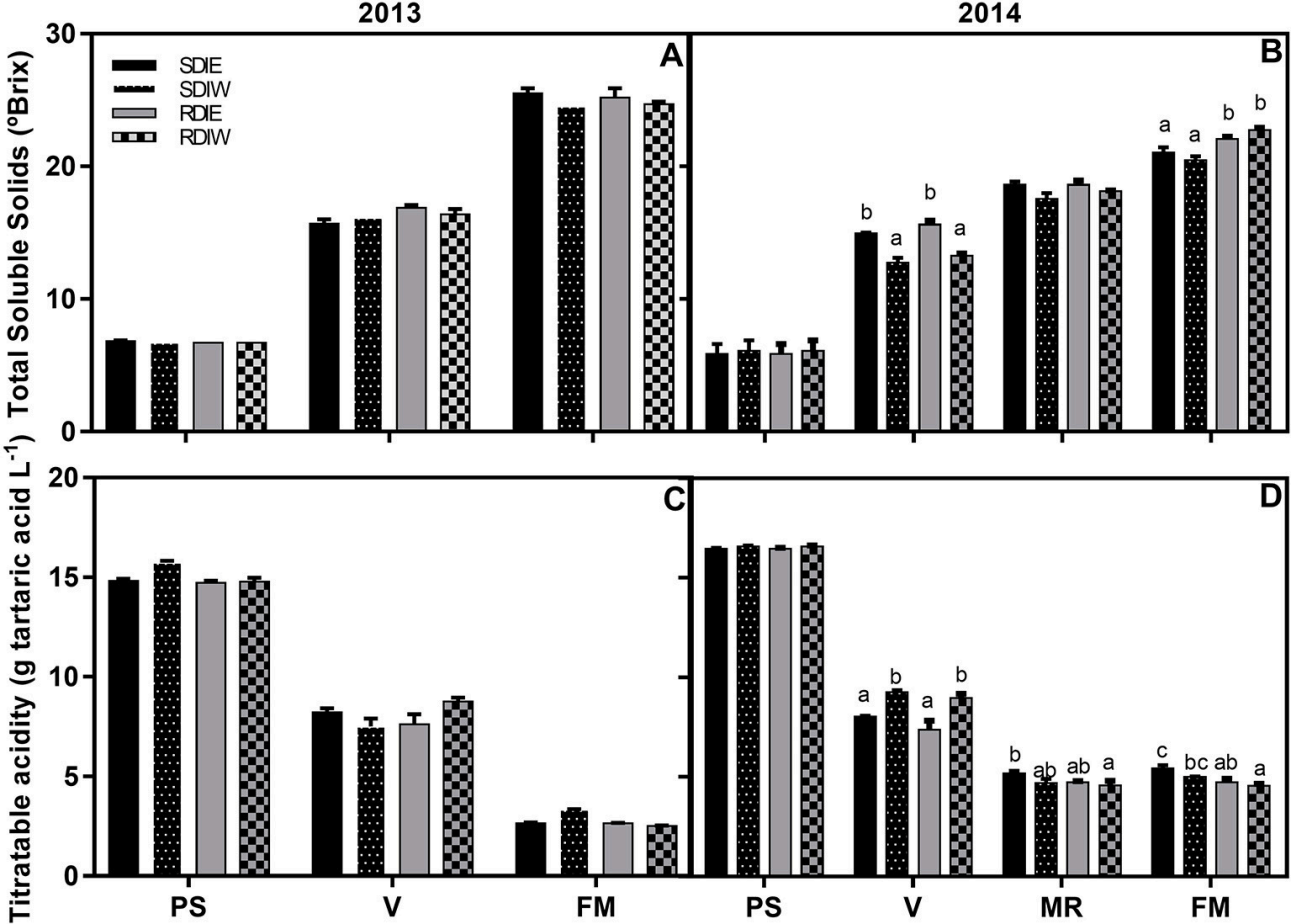
**Total soluble solids (TSS) (A,B)** and titratable acidity (TA) **(C,D)** in grape berries growing under sustained deficit irrigation (SDI) and regulated deficit irrigation (RDI) vines from two cluster position east (E) and west (W) in the seasons of 2013 and 2014. Values are mean ± SE (*n* ≥ 3). Different letters suffix indicate significant differences among treatments at the same date using Duncan test (*p* ≤ 0.05).

The trend of the TSS and TA accumulation both in 2013 and 2014 is presented in Figure 1. There was a significant season effect (*P* < 0.01) on both TSS and TA. TSS was higher in 2013 (25°Brix) than in 2014 (22°Brix), the reverse being observed for TA (~3.4 g L^−1^ in 2013 vs. ~4.9 g L^−1^ in 2014). In 2013, no significant TSS differences between irrigation treatments and side of the canopy were observed.

In 2014, differences between treatments and side of the canopy started to be observed at *véraison*. TSS starts to accumulate at *véraison* in berries from east-side followed by the west side both in SDI and RDI. This trend was also observed at mid-ripening, and at full maturation in SDI. At full maturation RDI presents higher TSS as compared with SDI, being highest at RDIW. The breakdown of TA was observed since *véraison* stage, especially in RDI. Differences in TA content were not observed between east and west berries at both pea size and *véraison* stages. On the contrary, from mid-ripening to full maturation, west side berries had lower TA content east side berries, in both SDI and RDI.

### Grape skin anthocyanin composition and accumulation

**Figure 2:**
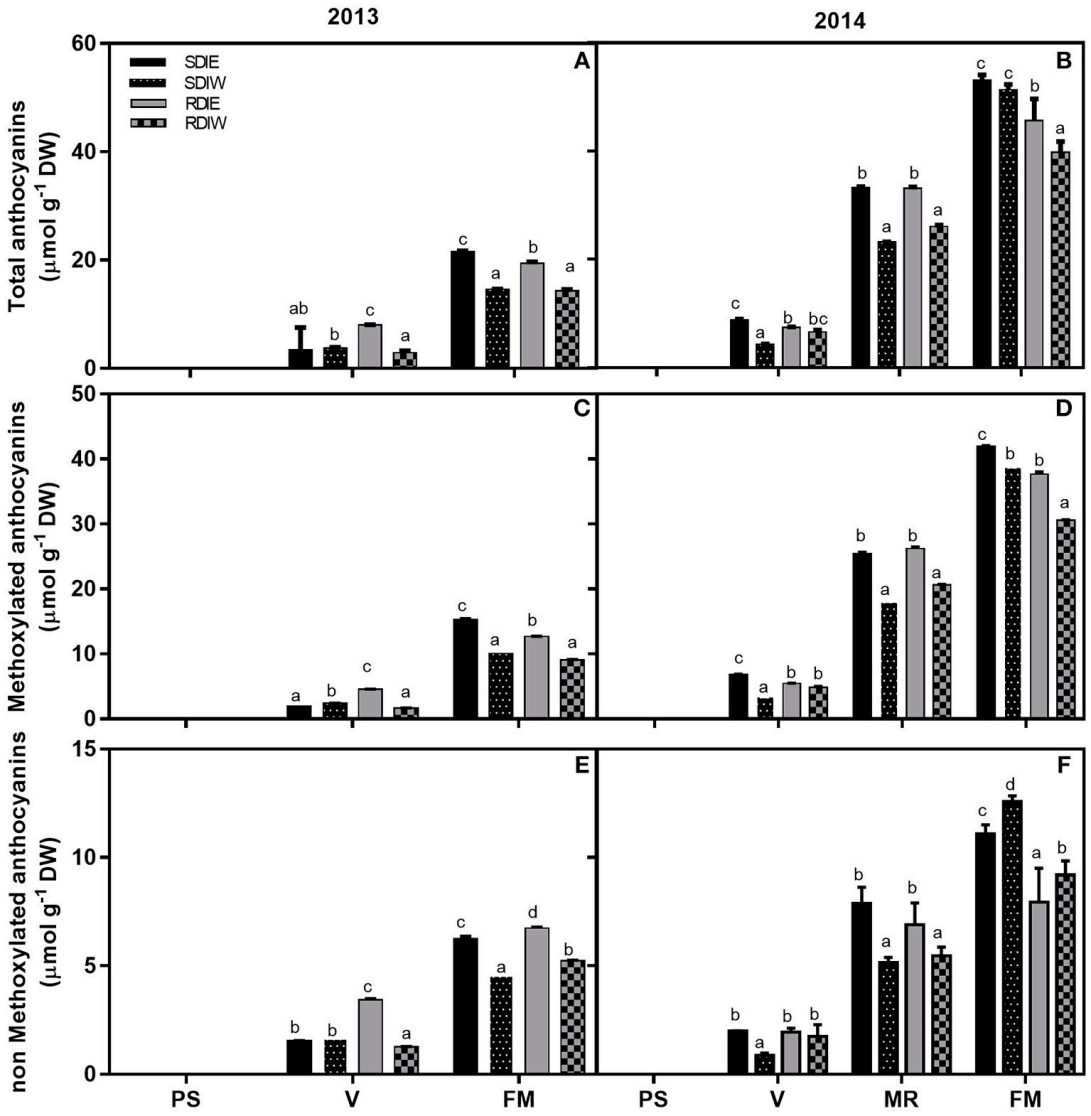
**Total anthocyanin (A,B)**, methoxylated **(C,D)**, and non-methoxylated **(E,F)** anthocyanin accumulation in grape berry skin growing under sustained deficit irrigation (SDI) and regulated deficit irrigation (RDI) vines from two cluster position east (E) and west (W) in the seasons of 2013 and 2014. Values are mean ± SD (*n* ≥ 3). Different letters suffix indicate significant differences among treatments at the same date using Duncan test (*p* ≤ 0.05).

A wide range of cyanidin, delphinidin, malvidin, petunidin, and peonidin derivatives were identified in either years of experimentation (Table 3), but their concentration was higher in 2014 than in 2013. Irrespective of the year, the concentration of anthocyanins increased since *véraison* to reach maximum levels at full maturation in all treatments (Figure 2). Anthocyanin levels were lower in RDI than SDI. West-exposed berries displayed lower anthocyanin concentration than east-exposed berries, irrespective of irrigation. In 2013, no differences in the trend of accumulation of anthocyanin were observed at *véraison*, except for RDIE which was significantly higher as compared with all other treatments. In contrast, at full maturation SDIE showed higher anthocyanin concentration as compared to SDIW and RDIW. Methoxylated anthocyanins (peonidin, petunidin, and malvidin) were highest in east exposed clusters in both irrigation regimes at full maturation (Figure 2). The ratio of methoxylated to non-methoxylated anthocyanins was the highest in RDIE, as well as in east sided than in west sided berries (Table 3).

**Table 3:** **Anthocyanin concentration in berry skins (μmol g^**−1**^ dry weight) of east- and west-exposed clusters under sustained deficit irrigation (SDI) and regulated deficit irrigation (RDI) along the berry development and during the seasons of 2013 and 2014**.

		***Véraison***				**Mid-ripening**				**Full maturation**			
		**SDIE**	**SDIW**	**RDIE**	**RDIW**	**SDIE**	**SDIW**	**RDIE**	**RDIW**	**SDIE**	**SDIW**	**RDIE**	**RDIW**
**ANTHOCYANINS (**μ**mol g**^−1^**DW)**													
Delphinidin-3-*O*-glucoside	2013	1.07 ± 0.04	1.17 ± 0.07	2.44 ± 0.20	0.86 ± 0.03	nd	nd	nd	nd	4.67 ± 0.42	3.28 ± 0.17	5.02 ± 0.18	3.77 ± 0.10
	2014	1.34 ± 0.05	0.56 ± 0.05	1.30 ± 0.09	1.08 ± 0.43	5.78 ± 0.52	3.43 ± 0.11	4.46 ± 0.84	3.54 ± 0.24	7.60 ± 0.35	8.58 ± 0.14	5.17 ± 1.03	6.23 ± 0.49
Cyanidin-3-*O*-glucoside	2013	0.36 ± 0.01	0.33 ± 0.01	0.74 ± 0.02	0.31 ± 0.03	nd	nd	nd	nd	0.58 ± 0.03	0.39 ± 0.03	0.87 ± 0.04	0.68 ± 0.02
	2014	0.30 ± 0.01	0.12 ± 0.02	0.32 ± 0.04	0.28 ± 0.02	0.80 ± 0.08	0.48 ± 0.01	0.53 ± 0.07	0.41 ± 0.05	0.84 ± 0.02	0.98 ± 0.03	0.68 ± 0.08	0.76 ± 0.07
Petunidin-3-*O*-glucoside	2013	0.59 ± 0.02	0.70 ± 0.06	1.47 ± 0.13	0.49 ± 0.02	nd	nd	nd	nd	3.40 ± 0.24	2.33 ± 0.12	3.41 ± 0.12	2.51 ± 0.05
	2014	1.11 ± 0.05	0.47 ± 0.05	0.99 ± 0.08	0.81 ± 0.37	4.61 ± 0.39	2.80 ± 0.08	3.83 ± 0.10	3.25 ± 0.24	6.31 ± 0.13	6.55 ± 0.09	4.61 ± 0.36	4.74 ± 0.24
Peonidin-3-*O*-glucoside	2013	0.29 ± 0.01	0.32 ± 0.01	0.65 ± 0.03	0.26 ± 0.02	nd	nd	nd	nd	0.95 ± 0.07	0.55 ± 0.03	1.01 ± 0.03	0.77 ± 0.02
	2014	0.58 ± 0.02	0.29 ± 0.05	0.58 ± 0.02	0.48 ± 0.04	1.51 ± 0.12	1.02 ± 0.01	1.24 ± 0.06	0.88 ± 0.06	1.77 ± 0.03	1.86 ± 0.05	1.85 ± 0.34	1.67 ± 0.02
Malvidin-3-*O*-glucoside	2013	0.85 ± 0.04	1.13 ± 0.11	1.96 ± 0.18	0.74 ± 0.03	nd	nd	nd	nd	7.79 ± 0.60	4.75 ± 0.22	5.95 ± 0.19	4.09 ± 0.07
	2014	3.52 ± 0.09	1.74 ± 0.23	2.73 ± 0.22	2.38 ± 1.05	13.26 ± 1.08	8.95 ± 0.28	13.76 ± 1.55	9.96 ± 0.42	21.80 ± 0.45	19.37 ± 0.42	18.45 ± 1.19	15.58 ± 0.16
Delphinidin-3-*O*-acetylglucoside	2013	0.03 ± 0.00	0.03 ± 0.00	0.07 ± 0.01	0.02 ± 0.00	nd	nd	nd	nd	0.16 ± 0.01	0.14 ± 0.01	0.15 ± 0.00	0.15 ± 0.00
	2014	0.00 ± 0.00	0.01 ± 0.00	0.03 ± 0.01	0.01 ± 0.00	0.20 ± 0.02	0.16 ± 0.00	0.20 ± 0.01	0.17 ± 0.02	0.29 ± 0.02	0.36 ± 0.00	0.25 ± 0.04	0.27 ± 0.02
Peonidin-3-*O*-acetylglucoside	2013	0.00 ± 0.00	0.00 ± 0.00	0.00 ± 0.00	0.00 ± 0.00	nd	nd	nd	nd	0.02 ± 0.01	0.03 ± 0.00	0.04 ± 0.00	0.03 ± 0.00
	2014	0.00 ± 0.00	0.00 ± 0.00	0.00 ± 0.00	0.00 ± 0.00	nd	nd	nd	nd	nd	nd	nd	nd
Malvidin-3-*O*-acetylglucoside	2013	0.03 ± 0.00	0.05 ± 0.01	0.08 ± 0.01	0.03 ± 0.00	nd	nd	nd	nd	0.52 ± 0.04	0.41 ± 0.02	0.36 ± 0.02	0.29 ± 0.01
	2014	0.25 ± 0.01	0.16 ± 0.03	0.19 ± 0.02	0.22 ± 0.08	0.97 ± 0.06	0.89 ± 0.08	1.28 ± 0.06	1.05 ± 0.00	1.96 ± 0.02	1.93 ± 0.05	1.98 ± 0.07	1.42 ± 0.03
Delphinidin-3-*O*-(6″-O-coumaroyl)glucoside (*cis* isomer)	2013	0.05 ± 0.01	0.07 ± 0.01	0.12 ± 0.01	0.05 ± 0.00	nd	nd	nd	nd	0.71 ± 0.08	0.60 ± 0.03	0.55 ± 0.03	0.50 ± 0.02
	2014	0.25 ± 0.01	0.12 ± 0.04	0.22 ± 0.03	0.26 ± 0.09	0.93 ± 0.11	0.94 ± 0.10	1.48 ± 0.05	1.16 ± 0.09	2.02 ± 0.05	2.26 ± 0.09	1.61 ± 0.37	1.66 ± 0.04
Cyanidin-3-*O*-(6″-O-coumaroyl)glucoside (*trans* isomer)	2013	0.04 ± 0.00	0.04 ± 0.00	0.07 ± 0.01	0.03 ± 0.00	nd	nd	nd	nd	0.13 ± 0.01	0.12 ± 0.01	0.15 ± 0.01	0.14 ± 0.01
	2014	0.09 ± 0.01	0.05 ± 0.00	0.08 ± 0.01	0.11 ± 0.01	0.17 ± 0.03	0.13 ± 0.02	0.23 ± 0.02	0.18 ± 0.00	0.33 ± 0.00	0.38 ± 0.01	0.24 ± 0.05	0.29 ± 0.02
Petunidin-3-*O*-(6″-O-coumaroyl)glucoside (*trans* isomer)	2013	0.04 ± 0.00	0.05 ± 0.01	0.10 ± 0.01	0.03 ± 0.00	nd	nd	nd	nd	0.60 ± 0.06	0.51 ± 0.02	0.49 ± 0.02	0.40 ± 0.01
	2014	0.23 ± 0.01	0.12 ± 0.01	0.18 ± 0.02	0.20 ± 0.08	0.92 ± 0.08	0.83 ± 0.02	1.18 ± 0.19	1.08 ± 0.04	1.73 ± 0.04	1.80 ± 0.02	1.41 ± 0.07	1.37 ± 0.03
Peonidin-3-*O*-(6″-O-coumaroyl)glucoside (*cis* isomer)	2013	0.00 ± 0.00	0.00 ± 0.00	0.01 ± 0.00	0.00 ± 0.00	nd	nd	nd	nd	0.01 ± 0.00	0.01 ± 0.00	0.01 ± 0.00	0.01 ± 0.00
	2014	nd	nd	nd	nd	nd	nd	nd	nd	nd	nd	nd	nd
Malvidin-3-*O*-(6″-O-coumaroyl)glucoside (*cis* isomer)	2013	0.01 ± 0.00	0.01 ± 0.00	0.01 ± 0.00	0.01 ± 0.00	nd	nd	nd	nd	0.05 ± 0.01	0.05 ± 0.00	0.05 ± 0.00	0.04 ± 0.00
	2014	0.07 ± 0.02	0.03 ± 0.00	0.04 ± 0.01	0.04 ± 0.01	0.18 ± 0.01	0.16 ± 0.02	0.24 ± 0.02	0.20 ± 0.01	0.23 ± 0.01	0.28 ± 0.03	0.29 ± 0.04	0.21 ± 0.03
Peonidin-3-*O*-(6″-O-coumaroyl)glucoside (trans isomer) + Malvidin-3-O-(6″-O-coumaroyl)glucoside (trans isomer)	2013	0.11 ± 0.01	0.15 ± 0.02	0.25 ± 0.03	0.09 ± 0.01	0.00 ± 0.00	0.00 ± 0.00	0.00 ± 0.00	0.00 ± 0.00	1.97 ± 0.15	1.44 ± 0.00	1.38 ± 0.08	0.96 ± 0.03
	2014	1.10 ± 0.04	0.59 ± 0.05	0.75 ± 0.07	0.71 ± 0.14	3.95 ± 0.32	3.38 ± 0.09	4.68 ± 0.26	4.21 ± 0.13	8.19 ± 0.16	6.94 ± 0.14	9.10 ± 0.93	5.59 ± 0.00
Methoxylated/non-Methoxylated Ratio	2013	1.50 ± 0.01	1.58 ± 0.01	3.31 ± 0.03	1.23 ± 0.01	nd	nd	nd	nd	5.54 ± 0.07	3.93 ± 0.04	6.18 ± 0.04	4.74 ± 0.02
	2014	3.46 ± 0.01	3.99 ± 0.02	2.83 ± 0.03	2.80 ± 0.10	3.23 ± 0.12	3.51 ± 0.03	3.80 ± 0.08	3.78 ± 0.07	3.79 ± 0.04	3.08 ± 0.03	4.75 ± 0.11	3.32 ± 0.07

*nd, non-detected; Values presented are mean ± SD (n ≥ 3)*.

In 2014 the level of anthocyanins since *véraison* stage was higher in east than in west-exposed barriers, irrespective of irrigation regime (Figure 2). This trend was even more evident at mid-ripening and at full maturation. Differences in anthocyanin concentration were not recorded between SDI and RDI at mid-ripening stage, whereas at full maturation RDI had lower anthocyanin content than SDI, irrespective of canopy position (Figure 2). The ratio of methoxylated to non-methoxylated anthocyanin ratio in SDI exceeded that in RDI at *véraison*, the reverse being observed at mid-ripening.

### Grape skin phenylpropanoids composition and accumulation

Different derivatives of hydroxycinnamic acid, such as caftaric and coutaric acid, and of quercetin and myricetin were identified in berry skin (Table 4). Traces of benzoic acids, e.g., gallic acid, protocatechuic acid, vanillic acid and syringic acid were also detected in 2013, but not in 2014. In general, phenolic acids (hydrocinnamic acids and benzoic acids) concentrations decreased along berry development and were higher in SDI as compared with RDI at all studied phenological stages, and in both seasons. In addition, phenolic acids were higher at west side than the east side at pea-size and *véraison*. The flavonol content was higher at west side than the east side (independent on irrigation treatment) since the pea size stage. The concentration of phenylpropanoids was greater in 2014 than in 2013.

**Table 4:** **Flavonol concentration in berry skins (μmol g^**−1**^ dry weight) of east- and west-exposed clusters under sustained deficit irrigation (SDI) and regulated deficit irrigation (RDI) along the berry development and during the seasons of 2013 and 2014**.

	**Pea-size**				***Véraison***				**Mid-ripening**				**Full maturation**				
		**SDIE**	**SDIW**	**RDIE**	**RDIW**	**SDIE**	**SDIW**	**RDIE**	**RDIW**	**SDIE**	**SDIW**	**RDIE**	**RDIW**	**SDIE**	**SDIW**	**RDIE**	**RDIW**
**FLAVONOLS (**μ**mol g**^−1^**DW)**																	
*trans*-caftaric acid	2013	5.73 ± 0.18	7.04 ± 0.39	6.00 ± 0.21	6.23 ± 0.15	2.50 ± 0.05	2.95 ± 0.07	2.04 ± 0.25	2.52 ± 0.07	nd	nd	nd	nd	1.40 ± 0.06	1.35 ± 0.03	1.13 ± 0.03	1.22 ± 0.04
	2014	8.26 ± 0.20	8.97 ± 0.20	8.26 ± 0.20	8.97 ± 0.20	3.48 ± 0.04	3.82 ± 0.27	3.80 ± 0.28	6.10 ± 0.71	2.45 ± 0.97	2.71 ± 0.27	2.44 ± 0.31	2.73 ± 0.17	2.31 ± 1.30	1.52 ± 0.12	1.70 ± 0.15	1.57 ± 0.01
Gallic acid + Protocatechuic acid + Vanillic acid + Syringic acid	2013	0.32 ± 0.02	0.38 ± 0.04	0.29 ± 0.00	0.30 ± 0.01	0.60 ± 0.04	0.61 ± 0.04	0.51 ± 0.01	0.53 ± 0.05	nd	nd	nd	nd	0.25 ± 0.02	0.20 ± 0.02	0.46 ± 0.02	0.39 ± 0.01
	2014	nd	nd	nd	nd	nd	nd	nd	nd	nd	nd	nd	nd	nd	nd	nd	nd
*trans*-coutaric acid	2013	5.72 ± 0.16	6.59 ± 0.51	5.63 ± 0.23	6.12 ± 0.13	2.85 ± 0.08	3.47 ± 0.10	2.23 ± 0.26	2.86 ± 0.19	nd	nd	nd	nd	1.70 ± 0.13	1.46 ± 0.10	1.35 ± 0.06	1.21 ± 0.09
	2014	7.58 ± 0.23	8.59 ± 0.17	7.58 ± 0.23	8.59 ± 0.17	2.97 ± 0.15	3.29 ± 0.33	3.48 ± 0.31	5.95 ± 0.31	2.17 ± 0.81	2.17 ± 0.81	2.35 ± 0.24	2.71 ± 0.57	2.20 ± 0.92	2.49 ± 1.31	1.45 ± 0.16	1.30 ± 0.12
Rutin + Quercetin-3-O-glucoside	2013	1.89 ± 0.12	1.85 ± 0.31	2.53 ± 0.03	2.50 ± 0.04	1.47 ± 0.04	1.32 ± 0.11	0.86 ± 0.04	1.19 ± 0.05	nd	nd	nd	nd	0.90 ± 0.05	1.34 ± 0.09	1.17 ± 0.09	1.30 ± 0.10
	2014	1.59 ± 0.32	2.44 ± 0.07	1.59 ± 0.32	2.44 ± 0.07	3.36 ± 0.04	2.57 ± 0.25	3.62 ± 0.39	3.85 ± 0.17	3.74 ± 0.32	2.07 ± 0.27	2.24 ± 0.35	2.11 ± 0.05	1.47 ± 0.24	2.77 ± 0.11	1.13 ± 0.15	2.84 ± 0.03
Myricetin 3-O-glucoside	2013	0.67 ± 0.05	0.54 ± 0.11	0.98 ± 0.04	0.90 ± 0.12	0.74 ± 0.02	0.63 ± 0.05	0.45 ± 0.05	0.64 ± 0.04	nd	nd	nd	nd	1.31 ± 0.12	1.45 ± 0.13	1.66 ± 0.12	1.48 ± 0.13
	2014	0.30 ± 0.16	0.57 ± 0.07	0.30 ± 0.16	0.57 ± 0.07	1.11 ± 0.03	0.88 ± 0.34	1.23 ± 0.12	1.27 ± 0.26	2.51 ± 0.16	1.08 ± 0.23	1.25 ± 0.24	1.26 ± 0.40	0.85 ± 0.07	3.63 ± 0.56	0.91 ± 0.35	2.97 ± 0.04

*nd, non-detected; Values presented are mean ± SD (n ≥ 3)*.

### Grape skin anthocyanin degradation products

**Figure 3:**
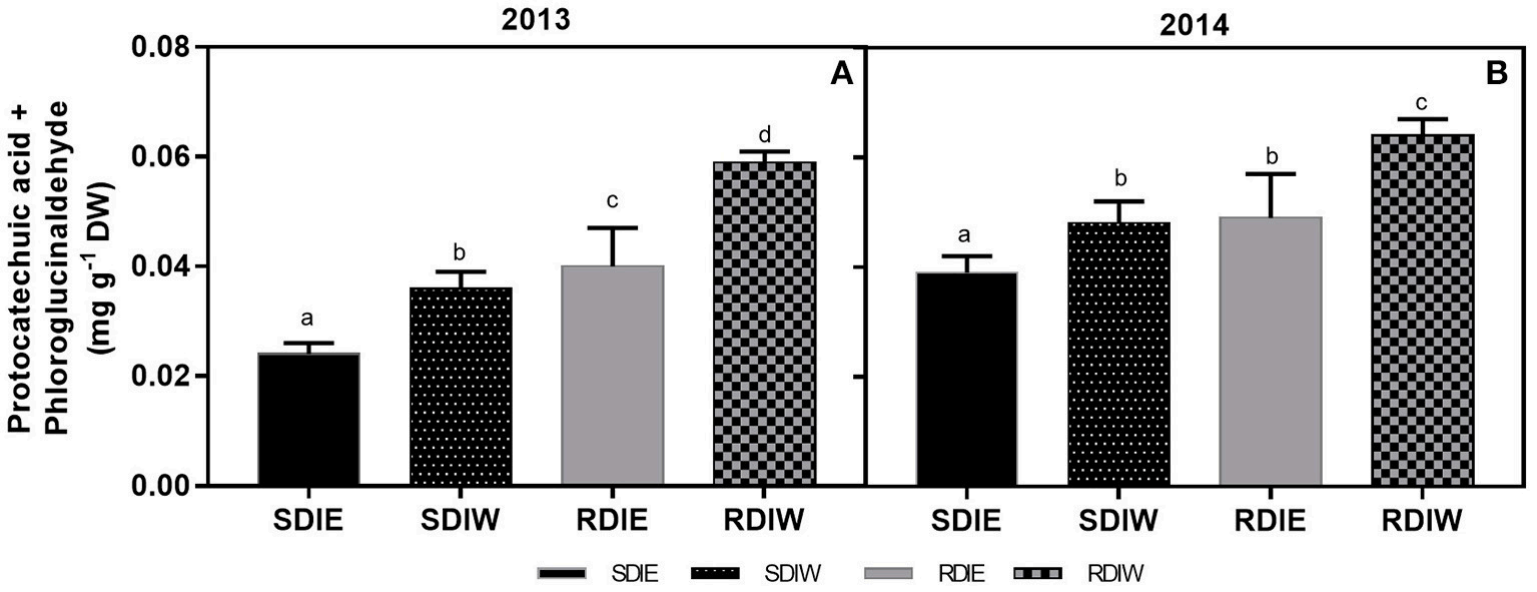
**Protocatechuic acid and phloroglucinaldehyde content (mg g^**−1**^ dry weight) in berry skin growing under sustained deficit irrigation (SDI) and regulated deficit irrigation (RDI) vines from two cluster position east (E) and west (W) in the seasons of 2013 (A)** and 2014 **(B)**. Values are mean ± *SD* (*n*≥3). Different letters suffix indicate significant differences among treatments at the same date using Duncan test (*p* ≤ 0.05).

Products of anthocyanin degradation, such as protocatechuic acid and phloroglucinaldehyde were only detected at full maturation (Figure 3), the concentration of which in west-exposed berries exceeded that in east-exposed berries. The degradation of anthocyanins was higher in berries under RDI.

### Free ABA and related metabolites accumulation in grape skin

**Figure 4:**
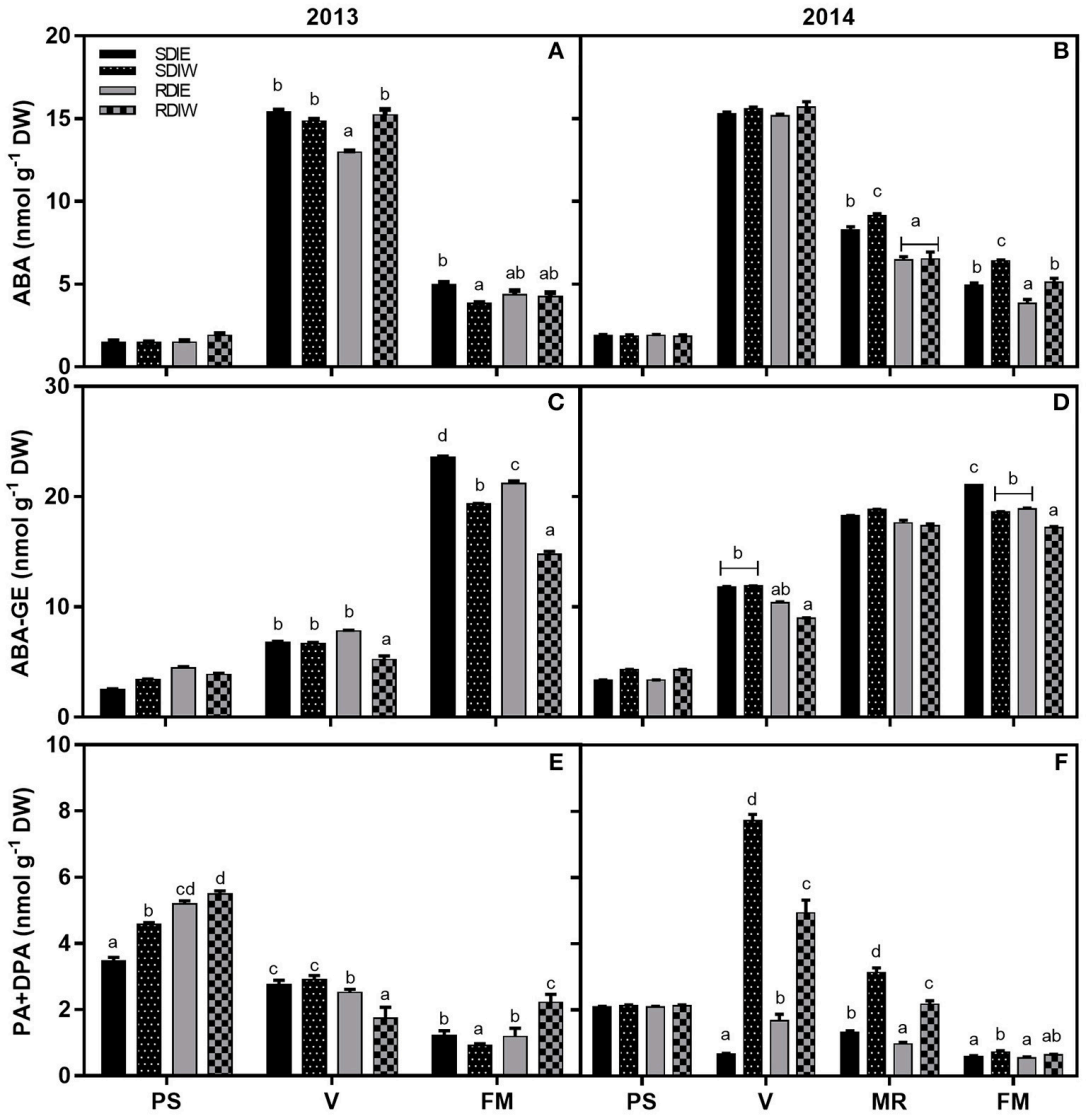
**Effect of irrigation regime: sustained deficit irrigation (SDI) and regulated deficit irrigation (RDI) and cluster position east (E) and west (W) on ABA (A,B)**, ABA-GE **(C,D)**, and PA+DPA **(E,F)** concentration in grape berry skins during the seasons of 2013 and 2014. Values are mean ± *SD* (*n*≥3). Different letters suffix indicate significant differences among treatments at the same date using Duncan test (*p* ≤ 0.05).

Free-ABA concentration sharply increased at *véraison* in both years (Figure 4). There were no significant differences at pea size and *véraison* in ABA skin concentration in 2014. In 2013, ABA was highest at west side independently from irrigation at pea size stage, and lowest in RDIE at *véraison*. In 2013, SDI treatment presented highest free ABA at full maturation stage, where east side presents highest values. In 2014, SDI treatment presented higher ABA both at mid-ripening and full maturation stages. In contrast with 2013, in 2014 west side presented the highest free ABA at mid-ripening and full maturation stages. DPA was the pre-dominant ABA catabolite present while PA concentration was significantly lower. PA and DPA concentrations showed the same trend of accumulation and decreased along berry ripening. During 2013, PA and DPA were highest at pea size and decreases thereafter. At this stage, RDI was higher than SDI and west side presented highest content. During 2014, no differences were observed at pea size, and PA and DPA showed a sharp increase at *véraison* stage in SDIW and RDIW while decreases at east side in both irrigation treatments. This highest content at west side was maintained at mid-ripening being SDIW with higher content than RDIW (Figure 4). Contrarily to PA and DPA, the ABA-GE increased along berry development in both years and its concentration was lower in RDI and at the west side.

### Expression of *VviUFGT, VviNCED1, VviβG1, VviHyd1*, and *VviHyd2* genes in grape berry skin

**Figure 5:**
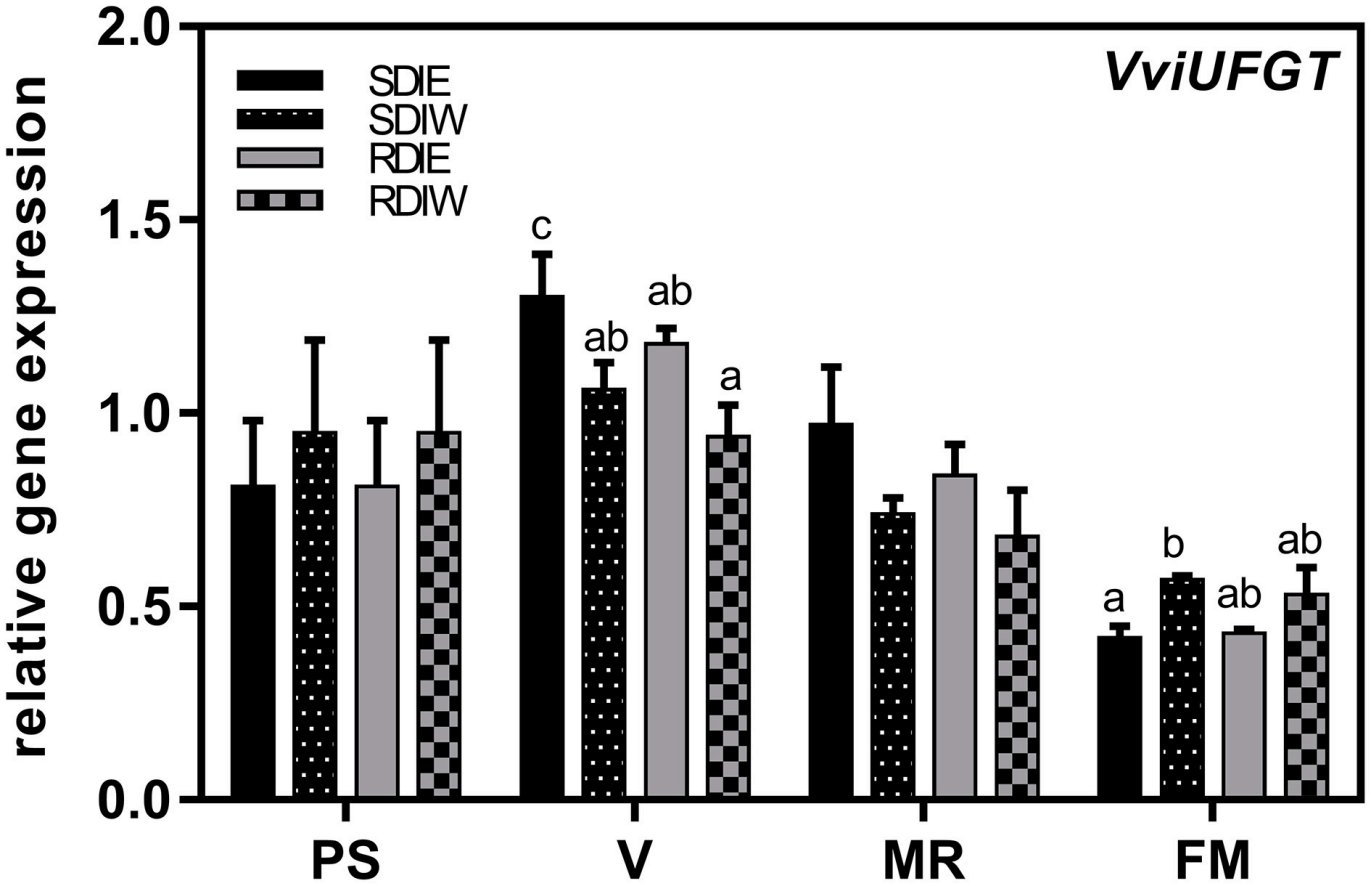
**Expression of the UDP-glucose:flavonoid 3-O-glucosyltransferase gene (***VviUFGT***), in berry skin growing under sustained deficit irrigation (SDI) and regulated deficit irrigation (RDI) vines from two cluster position east (E) and west (W) in the seasons of 2013 and 2014**. Values are mean ± SE (*n* ≥ 3). Different letters suffix indicate significant differences among treatments at the same date using Duncan test (*p* ≤ 0.05).

**Figure 6:**
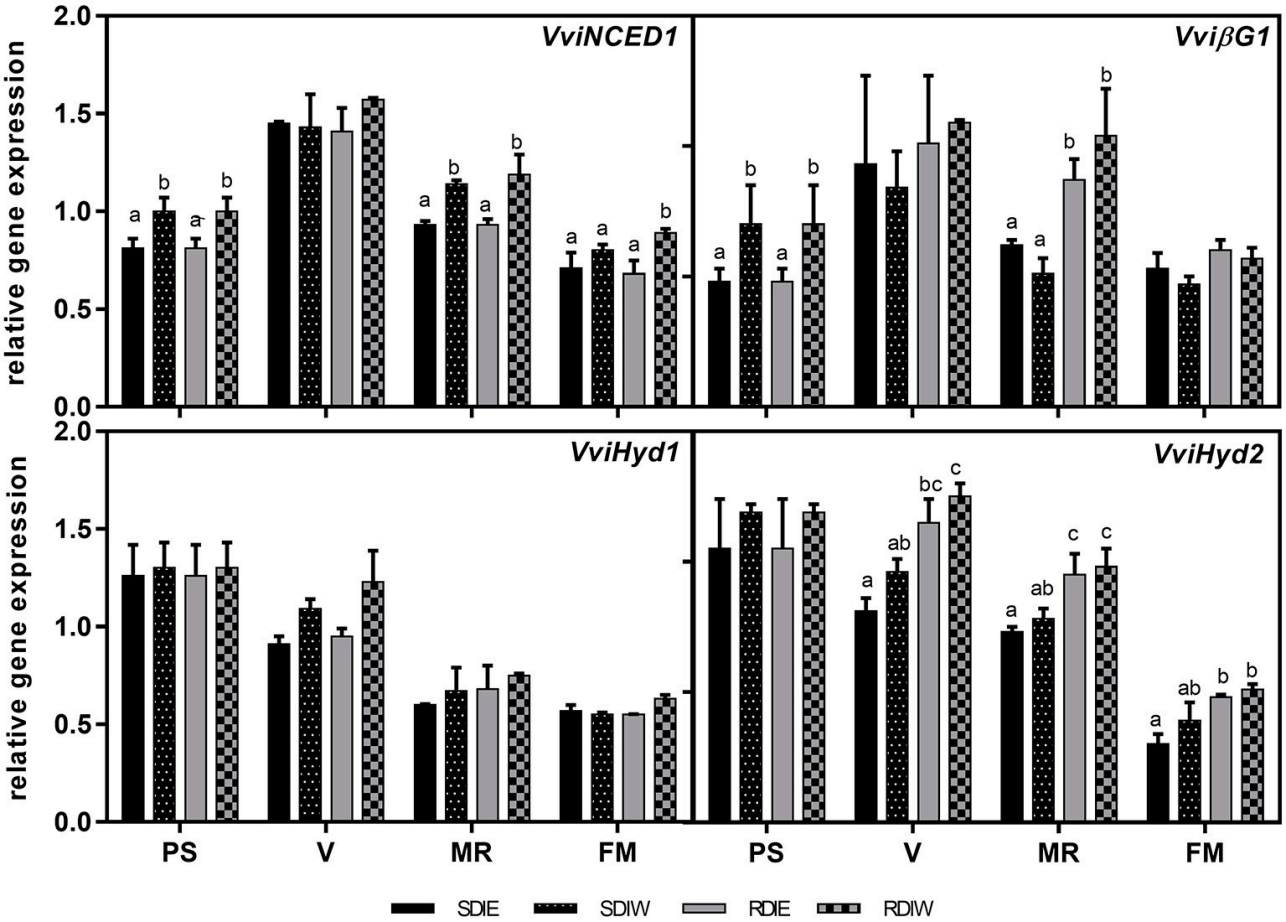
**Expression of several genes of the ABA pathway in berry skin growing under sustained deficit irrigation (SDI) and regulated deficit irrigation (RDI) vines from two cluster position east (E) and west (W) in the seasons of 2013 and 2014**. β-glucosidase (*Vv*β*G1*), 9-cis-epoxycarotenoid dioxygenase (*VviNCED1*), ABA 8′ hydroxylase 1 (*VviHyd1*) and ABA 8′ hydroxylase 2 (*VviHyd2*). Values are mean ± SD (*n* ≥ 3). Different letters suffix indicate significant differences among treatments at the same date using Duncan test (*p* ≤ 0.05).

The expression profile of *VviUFGT* was transiently up-regulated in all treatments at *véraison* stage during skin development (Figure 5). This increase in *VviUFGT* expression coincides with the onset of anthocyanin accumulation. Differences among treatments in the expression were observed from *véraison* until full maturation, where *VviUFGT* showed the maximum expression under SDI when compared with RDI. *VviUFGT* expression was also repressed at the west side both in SDI and RDI at *véraison*. However, at full maturation, *VviUFGT* was highest at SDIE while no differences due to the side were observed in RDI. *VviNCED1*, was studied at different phenological stages (Figure 6). A peak of expression was detected at *véraison* in both irrigation treatments. The expression of *VviNCED1* decreases from véraison onward, being more expressed at the west side of both irrigation treatments at mid-ripeningand at full maturation stages. In skin, *Vvi*β*G1* peaked at *véraison* stage. *Vvi*β*G1* expression was up-regulated at the west side of the canopy at pea size stage and in RDI at mid-ripening. Although no significant differences were recorded, a tendency in highest expression of *Vvi*β*G1* in RDI and at the west side of the canopy was recorded at *véraison*. The trend of expression of two genes encoding ABA 8′-hydroxylases (*VviHyd1* and *VviHyd2*) was studied along berry ripening (Figure 6). *VviHyd1* expression was higher at pea size stage but no statistical differences were recorded between east and west side of the canopy. Although, *VviHyd1* expression decreased thereafter, it maintained a statistical high expression level both in SDIW and RDIW at *véraison* coincident with high PA and DPA concentrations at this stage. *VviHyd2* presented a similar trend of expression than *VviHyd1*. However, *VviHyd2* was higher-expressed at the west side of the canopy at all phenological stages studied and in RDI treatment since *véraison* stage.

### Interaction between deficit irrigation and cluster position with berry ripening and quality traits

**Figure 7:**
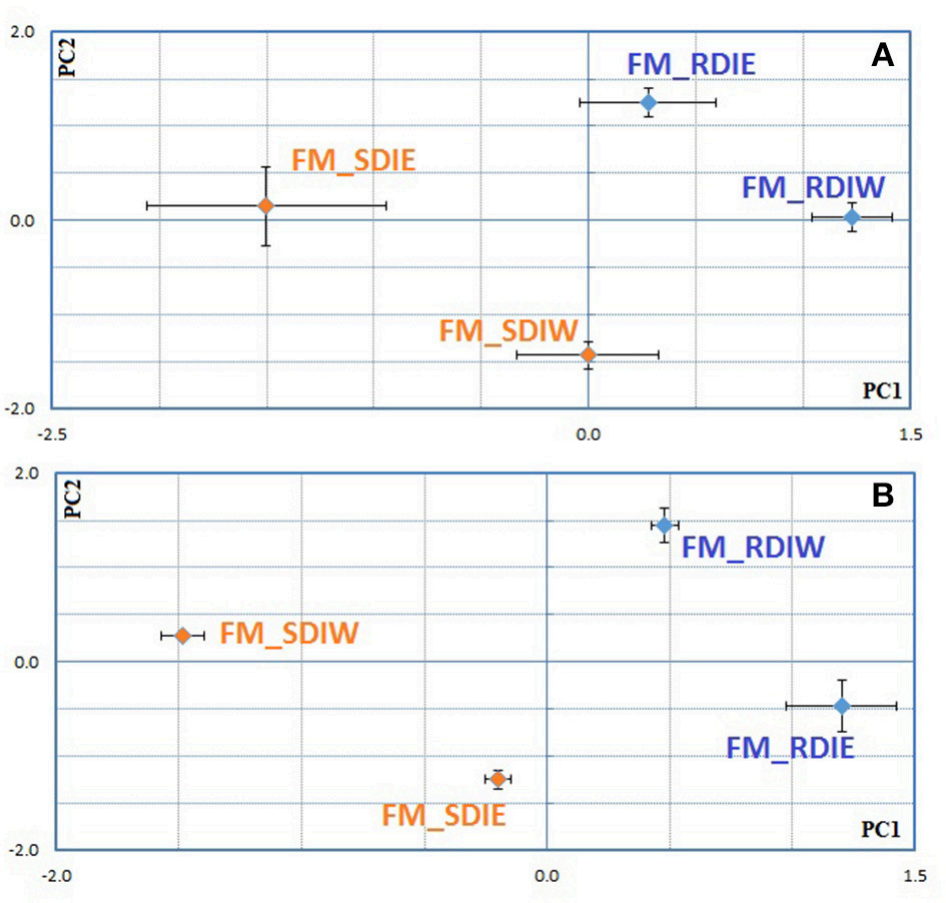
**Principal components analysis considering all data set from both growing seasons 2013–2014 (A)**, and individual growing season 2013 **(B)** and 2014 **(C)** for *Tempranillo* cv. at full maturation stage. **(A)** PC1 = 67%; PC2 = 13%; **(B)** PC1 = 50%; PC2 = 39%; **(C)** PC1 = 48%; PC2 = 26%. The plots were constructed using the row normed scores obtained with the ade4 package.

**Figure 8:**
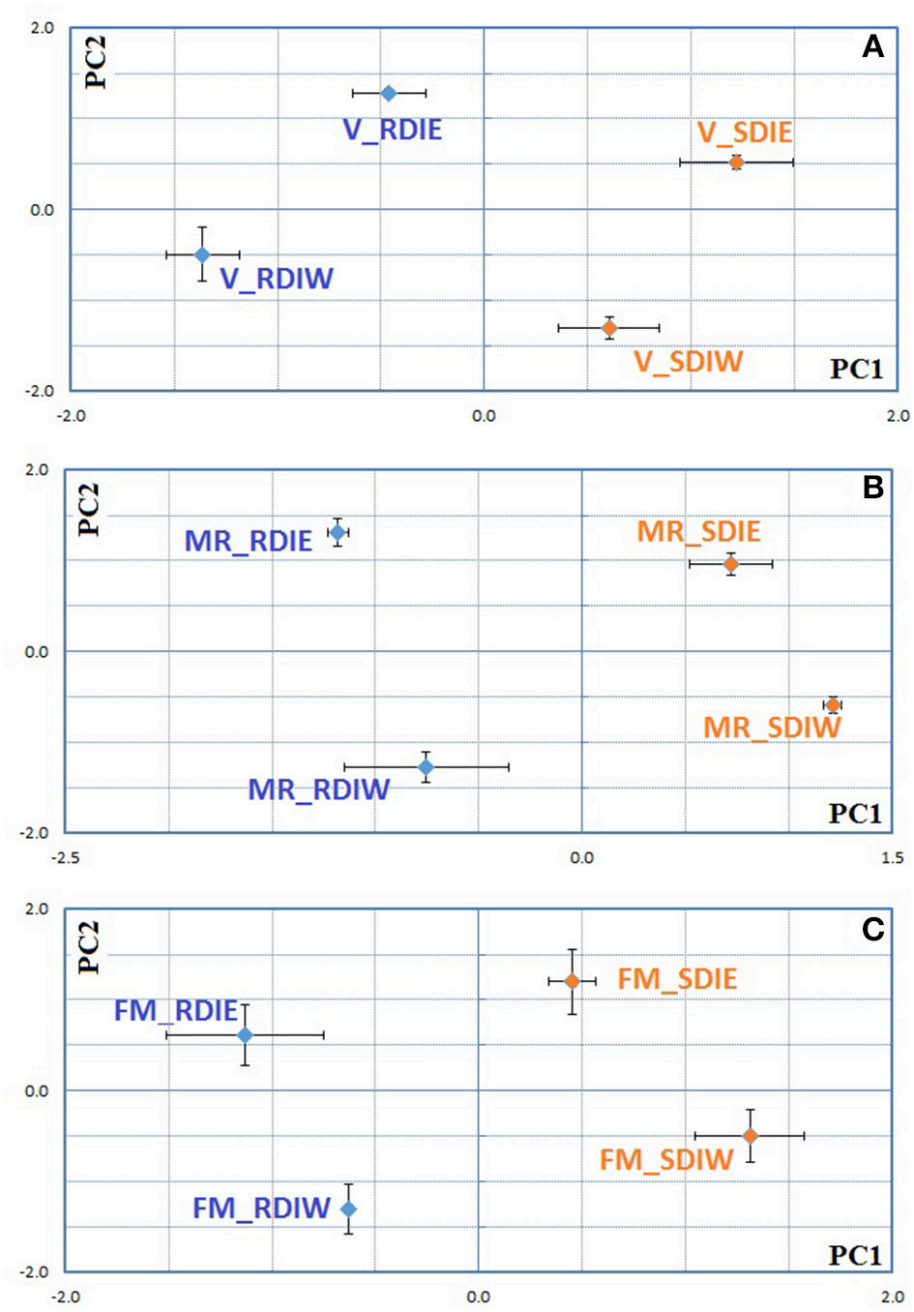
**Principal components analysis followed by between groups analysis considering ABA metabolites data set from 2014 growing seasons for Tempranillo cv. for véraison (A)**, mid-ripening **(B)**, and full maturation stages **(C)**. **(A)** PC1 = 39%; PC2 = 30% **(B)** PC1 = 44%; PC2 = 24% **(C)** PC1 = 39%; PC2 = 29%. The plots were constructed using the row normed scores obtained with the ade4 package.

PCA performed on cumulative water stress (SΨ_pd_), NHH and AEBT at *véraison* and full maturation separated the SDI from RDI as well as cluster position in both years (Supplementary Figure 2). In order to decipher the “vintage effect,” PCA was performed combining all data from both years (Figure 7A). This analysis showed that most traits contribute to the separation along the first axis and explain 67% of the variability (Supplementary Table 3), contributing thereby to a strong “vintage effect.” In addition, both deficit irrigation regime and cluster position, were discriminated along the 2nd and 3rd axis (13 and 11% respectively) in the 2 years (Figure 7A). Nonetheless, PCA showed difference between both studied years, being the impact of deficit irrigation regime greater in 2013, while in 2014 the cluster position had more influence (Figure 7A). ABA-GE parameter contributed for the separation along the 2nd and 3rd axis (Supplementary Table 3). Taking into consideration the data from Figure 7B (PCA for 2013) and 7C (PCA for 2014), we could further indicate that ABA-GE discriminated between deficit irrigation systems (Figure 7B, along 1st axis; Figure 7C, along 2nd axis) (Supplementary Table 3). In both cases, ABA-GE contributed for the separation SDI vs. RDI. However, only in 2014 it discriminates east vs. west cluster position. A distinctive response of ABA and ABA metabolism to both the irrigation treatment and the cluster position is observed in 2014 (Figure 8). It is important to note, that the first and the second axis explain 68–69% of observed variability and that the second axis contributes with at least a ½ to ¾ of the first axis.

Also for 2014, PCA shows (Figure 7C), that the parameters associated with cluster position discrimination (east vs. west) were the different chemical forms of malvidin (Malvidin-3-O-glucoside, Malvidin-3-O-acetylglucoside, and Peonidin-3-O-(6″-O-coumaroyl)glucoside + Malvidin-3-O-(6″-O-coumaroyl)glucoside), flavonols (rutin and quercetin-3-O-glucoside and myricetin), and degradation products of anthocyanins. These molecules, in a year with low water availability (high SΨ_pd_), were able to discriminate east-west position and responded to the berry temperature. In contrast, in 2013, the separation according to cluster position was only achieved along the 4th axis (7%) and none of above mentioned parameters contributed significantly for such separation (Supplementary Table 3). This indicates that in 2013, other factors in addition to water stress and T_berry_ are impacting on berry skin metabolism. It appears that in wet years, vines could cope with high T_air_ in a way that made difficult to separate the effects of irrigation from cluster position.

In order to explain the effect of water stress and/or cluster position on berry ripening and composition, we explore for the existence of correlations. To our surprise, neither anthocyanins nor ABA correlated with SΨ_pd_ in both years. Nonetheless, a highly significant and negative correlation between free-ABA and total anthocyanins was observed for both irrigation treatments (*r* = −0.76, *p* ≤ 0.0001) in the 2 years. Interestingly, a weaker correlation was observed on the west side as compared with the east side (east *r* = −0.84, *p* ≤ 0.001 vs. west *r* = −0.67, *p* ≤ 0.05) indicating that high temperature affect negatively ripening process, namely anthocyanin accumulation. In addition, a thermal disruption of the anthocyanins/ABA relationship among the different irrigations and cluster positions was observed, being the RDIW showing the larger difference, suggesting the higher susceptibility of RDI to high temperature as compared with SDI.

## Discussion

Ripening is a complex process leading to several physiological and metabolic changes including softening, and sugar and anthocyanin accumulation. In contrast to climacteric fruits in which the control of ripening is well established, in non-climacteric grape berries, ripening is, still, poorly understood.

Hormones, particularly ABA, are long known to participate in the ripening process of grape berry (Jeong et al., 2004; Symons et al., 2006; Wheeler et al., 2009; Koyama et al., 2010; Sun et al., 2010; Böttcher and Davies, 2012). Deficit irrigation has been proposed as suitable viticultural practice to influence berry ripening, through the stimulation of sugar and anthocyanin biosynthesis under a mild to moderate water deficits (Castellarin et al., 2007a; Gambetta et al., 2010). However, deficit irrigation has been shown to increase (Basile et al., 2011; Santesteban et al., 2011) decrease (Intrigliolo et al., 2012; Zarrouk et al., 2012) or to have negligibly affect (Castellarin et al., 2007a,b; Girona et al., 2009) on anthocyanin biosynthesis. Mechanisms through which water deficit affects berry ripening require further elucidation, mostly because increases in sugars and anthocyanins in berries exposed to pre- or post-véraison deficit irrigation depends greatly on the sensitivity of berry developmental growth and cultivar's sensitivity to ABA signaling (Ferrandino and Lovisolo, 2013).

Air temperature, namely high temperatures, may also have a strong impact on berry quality, leading to a drop in berry acidity and an increase in sugar content (Keller, 2010), while reducing anthocyanin content (Mori et al., 2007; Tarara et al., 2008; Greer and Weedon, 2013) and varietal aroma (Jones and Goodrich, 2008; Bonada et al., 2015). Our study aimed at evaluating the combined effect of changes in water availability and berry temperature (by sampling berries located on different sides in the canopy) on grape berry ripening and composition.

### Interactive effects of irrigation regime and berry temperature on the biosynthesis, accumulation, and degradation of anthocyanins

Water deficit has been considered to impact on the accumulation of anthocyanins through the stimulation of anthocyanin hydroxylation (Castellarin et al., 2007b), which converts hydroxylated anthocyanins (cyanidin and delphinidin) into their methoxylated derivates (peonidin, petunidin, and malvidin) (Kennedy et al., 2002; Castellarin et al., 2007a,b). In contrast, high temperature was shown to reduce anthocyanin hydroxylation in grape berries (Tarara et al., 2008). Our study shows that methoxylated anthocyanins accumulated from mid-ripening to full maturation stages in berries located on the east canopy side, irrespective of year and irrigation regime. The ratio of methoxylated to non-methoxylated anthocyanin was higher in RDI than SDI vines, as reported previously (Castellarin et al., 2007a,b; Deluc et al., 2009). On the other hand, the ratio of methoxylated to non-methoxylated anthocyanins was higher in berries located at the west than at the east side, confirming previous findings of high temperature influence on the anthocyanin forms (Tarara et al., 2008).

Methoxylation of anthocyanins greatly affects berry color and stability (Jackman and Smith, 1996). Our data suggests that anthocyanin methoxylation might represent a strategy adopted by grapes to cope with the combined effects of water shortage and heat stress. Increasing anthocyanin stability through methoxylation may partly compensate for reduced anthocyanin biosynthesis when carbon assimilation is severely constrained under severe stress (i.e., the combined effect of water deficit and heat stress). The concentration of anthocyanins was significantly repressed in west-exposed berries, both in SDI and RDI during ripening in 2013. In 2014, no differences in anthocyanin concentrations between sides were observed in SDI at full maturation suggesting that more irrigated SDI vines are less sensitive to high temperatures during ripening in coolest years or/and years with less hours of extreme temperature which corroborates recent studies of Romero et al. (2016). In contrast, RDI showed statistically lower concentration at the west side than at the east side and lower than SDI. Our findings suggest that the most severe stress conditions, i.e., RDI treatment, exacerbated the negative impact of high temperature on anthocyanin biosynthesis and/or degradation. Our hypothesis is further corroborated by the observation that the expression of *VviUFGT*, which is involved in late steps of anthocyanin biosynthesis, was down-regulated in RDI as compared to SDI at the onset of ripening. This also supports the negative effect of higher pre-*véraison* SΨ_pd_ on anthocyanin biosynthesis. The expression of *VviUFGT* was also repressed at the west side in both irrigation treatments, possibly due to greater heat stress incidence in this side (Table 2). In contrast, *VviUFGT* expression was higher at west side than in east side in both treatments at full maturation, suggesting the possibility of an enhancement of anthocyanin biosynthesis at this stage. However, this enhancement was not translated into higher anthocyanin concentrations in the berries located at the west side. This may be due to the highest rate of degradation registered on the west side of the canopy in both irrigation treatments (Figure 4). Our results suggest that the negative impact of water stress and high temperature on anthocyanin content likely results from the repression of anthocyanin biosynthesis at the onset of ripening. We also provide evidence that accumulation of anthocyanins at full maturation is negatively affected by water/heat stress possibly because of the higher degradation rate of these compounds at later stages of berry ripening.

Interactive effects of water deficit and side of the canopy on berry quality are clearly exposed in our study. There is compelling evidence that the negative impact of water deficit on berry quality greatly depends on temperature (Tarara et al., 2008; Bonada et al., 2013; Fernandes de Oliveira and Nieddu, 2013). In our experiment, an annual effect on anthocyanin accumulation was observed, 2013 exhibiting lower concentrations than in 2014, in spite of 2013 showing lower accumulated water stress (SΨ_pd_). The high temperatures observed in the growing season of 2013 (2°C higher than 2014) could explain these results. On the other hand, in 2013, berries accumulated more hours with T_air_ above the threshold of >35°C during the ripening period, as compared with 2014. This could explain the acceleration of sugar accumulation (TSS) and acid breakdown (TA) observed in 2013 with regards to 2014. While TSS was higher in 2013 than in 2014 at full maturation, anthocyanins were lower in 2013. This result corroborates the fact that high temperature promotes sugar concentration in detriments of other quality attributes of berries, leading to poorly balanced wines with reduced varietal characteristics (Mira de Orduña, 2010). Sugar accumulation was reported to be required for anthocyanin biosynthesis in grape berries (Gollop et al., 2002; Castellarin et al., 2007a). Although no differences were observed in TSS due to irrigation treatments and cluster position in 2013, anthocyanins were repressed at the west side of the canopy. In 2014, TSS was lower at west side at *véraison*, which corroborates with low anthocyanin concentration results. However, at full maturation, TSS was higher in RDI (both sides of the canopy) as compared with SDI, while anthocyanins were lower in RDI treatments (east and west). Taking into account the more stressful temperature conditions in 2013, and higher cumulative heat stress from pea size to full maturation at west exposure in 2014, these results suggest a decoupling of anthocyanin/sugar as a consequence of high temperature (Sadras and Moran, 2012; Bonada et al., 2013).

### Differential regulation of flavonol and other phenylpropanoid accumulation during berry development by irrigation and cluster position

The influence of water and heat stress on flavonol profile in grapes is still unclear, in contrast with compelling evidence reported for anthocyanin biosynthesis (Ferrandino and Lovisolo, 2013). Our study offers the first evidence that the combined effect of water and temperatures stress is distinct from those reported for water- or heat stressed grapes alone (Spayd et al., 2002; Castellarin et al., 2007a,b; Azuma et al., 2012; Zarrouk et al., 2012).

Deficit irrigation did not enhance the concentration of flavonol compounds, and no differences were observed between SDI and RDI berries. The data corroborates Deluc et al. (2009), which showed that flavonol content is not affected by water stress in a red grapevine variety but it is in a white one. Nevertheless, flavonols were higher at the west side of both SDI and RDI treatments, suggesting an enhancement of their accumulation/biosynthesis under heat stress. The results suggest that the same climactic conditions that influence anthocyanin biosynthesis/accumulation at west side of the canopy may also affect flavonols since they share the same biosynthetic pathway. In this sense, the present study may suggest that the increase of flavonol concentration is promoted by the decrease in the UDP-glucose:flavonoid 3-O-glucosyltransferase activity by heat stress. We found that the transcript of this enzyme (*VviUFGT*) is down-regulated, suggesting that more substrate is available for flavonol biosynthesis via flavonol synthase activity. The competition for substrates (that both enzymes share) supports it. These results show also the differential impact of irrigation is dependent upon the stage of berry growth and the year, and suggest the multiple roles of flavonols during development, as previously reported by Cohen et al. (2008). In addition, the accumulation of phenolic acids at west side, may account for a protective effect against oxidative stress thanks to their chemical structures. Phenolic acids are able to scavenge free radicals (Blokhina et al., 2003), which are expected to be generated by high temperatures.

### Water deficit and cluster position modulate ABA metabolism in the berry

ABA is a key signal in the trigger of berry ripening, as suggested by the increase of berry ABA content around *véraison* (Wheeler et al., 2009; Giribaldi et al., 2010; Sun et al., 2010; Zifkin et al., 2012; Karppinen et al., 2013; Niculcea et al., 2013). However, the effect of water stress on ABA concentration in grape berries is far from being conclusively addressed (Deluc et al., 2009; Balint and Reynolds, 2013; Niculcea et al., 2013, 2014). In addition, contrasting findings have been reported in studies exploring the effect of heat stress on ABA metabolism (Azuma et al., 2012; Carbonell-Bejerano et al., 2013; Rienth et al., 2014) which appear to be particularly dependent on grape berry phenology. In this sense, Rienth et al. (2014) showed that ABA synthesis was repressed by heat stress at the beginning of *véraison* and enhanced at the end of this developmental stage.

In our study, free-ABA concentration increased at *véraison* in both years, concomitantly with the sharp increase in transcripts of ABA biosynthesis genes, *VviNCED1* and *Vvi*β*G1*. This enhancement of ABA-biosynthetic genes coincided with a steep increase in berry sugars and anthocyanins. Irrigation regime and berry position had little effect on free-ABA levels at *véraison*, in contrast with their effects on sugar and anthocyanin. Additionally, although the strong correlation found between free-ABA and total anthocyanins on both sides of the canopy, the west side showed a weaker correlation as compared with the east side. We hypothesize that high temperature account for the differential modulation of these metabolites, since it was demonstrated to uncouple some ripening-related traits (Sadras and Moran, 2012; Carbonell-Bejerano et al., 2013; Sadras et al., 2013).

The biological function of ABA in grape ripening requires a transient increase at *véraison* as well as the subsequent decrease, which seems to be under developmental control (Wheeler et al., 2009). This hypothesis is reinforced by our study since ABA concentration in skins was found to not respond to vine water status (SΨ_pd_). On the other hand, the ABA increase coincides with the decrease in the PA and DPA, suggesting that this increase is caused by a decreased catabolism rate (Castellarin et al., 2015) and increased biosynthesis. Our results also point out to the effect of environmental conditions on ABA catabolism, since effects of irrigation and cluster position on PA and DPA were recorded. The molecular regulation of PA and DPA shows a complex pattern. The genes encoding ABA 8′-hydroxylases (*VviHyd1* and *VviHyd2*) were expressed equally in berry skin, but only *VviHyd2* was modulated by water stress, being up-regulated under RDI treatment.

The decrease of ABA after *véraison* may be due to several processes of degradation and oxidation. ABA-GE increased from pea size, reaching maximum content at full maturation, being higher in SDI treatment and repressed in west side of the canopy. This suggests that ABA-GE responds negatively to high temperatures and water stress intensity, contrasting with previous reports analysing ABA-GE under water stress (Deluc et al., 2009; Zarrouk et al., 2012; Balint and Reynolds, 2013). This highlights the need to carefully monitor water stress imposition and progression in order to be able to compare data sets originated from distinct experimental setups.

Grape berry can also modulate it ABA concentration via the release of ABA-GE by β-glucosidase (Lee et al., 2006; Sun et al., 2010; Zhang et al., 2013). *Vv*β*G1* transcript increased around *véraison*, coincident with ABA accumulation, which indicates that β-glucosidase might play a role in regulating the level of ABA during the ripening at this stage. At mid-ripening, *Vv*β*G1* transcript was up-regulated in RDI skins, suggesting a high release of free ABA from ABA-GE in this treatment. This latter result may also explain in part the lower ABA-GE concentrations at full maturation in RDI. Altogether, these results indicate that ABA found in skin along berry development may be also derived from the de-conjugation process of ABA-GE at later stages of berry development. These findings support a role for β-glucosidase in the modulation of ABA content during grape berries development and in response to dehydration.

The accumulation pattern of the different ABA related products provided evidence that endogenous ABA content is modulated by a dynamic balance between biosynthesis and catabolism. It appears that in grape berry, the ABA homeostasis is achieved by degradation before *véraison*, while after *véraison* ABA homeostasis is realized by conjugation. In addition, changes in ABA catabolism/conjugation along berry development were affected by water stress particularly when also submitted to heat stress, indicating that ABA-GE and ABA catabolites play an essential role in ABA homeostasis under environmental constraints.

## Conclusion

Our work shows that the impact of irrigation regime and high temperature interaction in the control of berry ripening is quite complex and dynamic. Multivariate analysis showed that the strongest parameter influencing the berry ripening is the deficit irrigation system, in spite of some differences observed between the 2 years. Nonetheless, berry temperature is an important variable conditioning the deficit irrigation effect on berry quality. Deficit irrigation has a positive effect on berry composition only when the high temperature is not a limiting factor. Seasons with increased water stress, lead to a larger impact of high temperatures on the berry ripening and composition. Our results also show that the negative impact of water stress and high temperature on anthocyanins results from the repression of biosynthesis at the onset of ripening and from degradation at later stages Independently from the effect of water stress and cluster position, the increase in free ABA took place at the same stage as anthocyanin and sugar accumulation in the skin. Water and heat stresses do not affect free ABA, but do alter ABA catabolism/conjugation. This suggests that the homeostasis of berry ABA under abiotic stresses is mostly controlled by of catabolism/oxidation processes. Besides the observed *season*-specific effect on the accumulation and biosynthesis of different metabolites in the grape berry, and in general, SDI gives rise to berries with greater concentrations of phenolics than RDI, with the additional advantage of attenuating heat incidence at the west side of the canopy.

## Author contributions

OZ and MC conceived and planned the study. CB, AG, and MT performed the HPLC analysis of flavonoids and ABA. CB and MT performed the data integration and processing for the above compounds. RE and CL implemented and maintained the viticultural treatments and monitored the vineyard. RE did the climatic and irrigation data processing and analysis. CP performed PCAs data analysis. OZ and TG did the sampling and the processing and analysis of the berry samples. OZ and CP performed data analysis. OZ, CP, and MC drafted the initial manuscript, all authors contributed to the final manuscript.

### Conflict of interest statement

The authors declare that the research was conducted in the absence of any commercial or financial relationships that could be construed as a potential conflict of interest.
