# Supplementary material for: Grape Ripening Is Regulated by Deficit Irrigation/Elevated Temperatures According to Cluster Position in the Canopy
Source: Front Plant Sci. 2016 Nov 15;7:1640. doi: 10.3389/fpls.2016.01640 (PMC5108974; doi:10.3389/fpls.2016.01640)
Supplement: Supplementary file 1 [file Table1.PDF]

**Supplementary Table 1.** Irrigation water applied (mm) in sustained deficit irrigation (SDI) and regulated deficit irrigation (RDI) treatments during the period pea size - *véraison* (PS-V), *véraison*-mid-ripening (V-MR), f mid-ripening-full maturation (MR-FM) and from *véraison*-full maturation (V-FM) in 2013 and 2014 growing seasons.

| | | IRRIGATION (mm) | | | |
|---|---|---|---|---|---|
| | | DOY | | | |
| | | ini | end | SDI | RDI |
| 2013 | PS - V | 191 | 210 | 21.3 | 6.6 |
| | V - FM | 211 | 230 | 33.0 | 19.8 |
| | Total | | | *54.3* | *26.4* |
| 2014 | PS - V | 168 | 195 | 11.5 | 6.0 |
| | V - MR | 196 | 210 | 11.5 | 11.5 |
| | MR - FM | 211 | 223 | 12.0 | 9.0 |
| | Total | | | *35.0* | *26.5* |
