# Supplementary material for: Grape Ripening Is Regulated by Deficit Irrigation/Elevated Temperatures According to Cluster Position in the Canopy
Source: Front Plant Sci. 2016 Nov 15;7:1640. doi: 10.3389/fpls.2016.01640 (PMC5108974; doi:10.3389/fpls.2016.01640)
Supplement: Supplementary file 2 [file Table2.PDF]

Supplementary Table 2. Rainfall, Average (T_{air}_avg), Maximum (T_{air}_max) and Minimum (T_{air}_min) air temperature and Number of hours with temperature below 10 ºC (T_{air} < 10ºC) and above 35 ºC (T_{air} > 35ºC) during the period pea size-*véraison* (PS-V), *véraison*-mid-ripening (V-MR), mid-ripening-full maturation (MR-FM) and *véraison*-full maturation (V-FM) in 2013 and 2014 growing seasons.

YEAR	PHENOLOGY	Rainfall (mm)	T_{air}_avg (ºC)	T_{air}_max (ºC)	T_{air}_min (ºC)	T_{air} < 10 ºC (hours)	T_{air} > 35 ºC (hours)
2013	PS - V	0.2	24.1	38.8	13.0	0.0	27
	V - FM	0.0	26.2	41.9	12.4	0.0	94
	PS - FM	0.2	25.3	41.9	12.4	0.0	121
2014	PS - V	11.6	21.9	38.1	11.4	0.0	36
	V - MR	0.0	24.3	42.3	13.3	0.0	43
	MR - FM	0.0	23.7	36.4	12.7	0.0	4
	PS - FM	11.6	23.0	42.3	11.4	0.0	83
