# Supplementary material for: Grape Ripening Is Regulated by Deficit Irrigation/Elevated Temperatures According to Cluster Position in the Canopy
Source: Front Plant Sci. 2016 Nov 15;7:1640. doi: 10.3389/fpls.2016.01640 (PMC5108974; doi:10.3389/fpls.2016.01640)
Supplement: Supplementary file 3 [file Table3.PDF]

Supplementary Table 3: The Pearson's product correlation coefficient was calculated in order to disclose significant relationships between principal componentes and the variables analyzed considering all data set for Tempranillo cv. at full maturation stage

Correlation coefficients related to Figure 7A

	Axis1	*p*- value	Axis2	*p*- value	Axis3	*p*- value	Axis4	*p*- value
**Spredawn**	-0.81	0.000	-0.37	ns	-0.21	ns	-0.35	ns
**NHH**	-0.67	0.000	-0.33	ns	-0.21	ns	-0.42	0.041
**Delphinidin-3-O-Glc**	0.90	0.000	-0.10	ns	0.33	ns	-0.20	ns
**Cyanidin-3-O-Glc**	0.62	0.001	-0.38	ns	0.26	ns	-0.59	0.002
**Petunidin-3-OGlc**	0.96	0.000	0.04	ns	0.18	ns	-0.18	ns
**Peonidin3-O-Glc**	0.93	0.000	-0.03	ns	-0.14	ns	-0.17	ns
**Malvidin-3-O-Glc**	0.97	0.000	0.17	ns	-0.09	ns	-0.10	ns
**Delphinidin-3-O-acetateGlc**	0.97	0.000	-0.14	ns	0.12	ns	-0.03	ns
**Peonidin3O-acetylGlc**	0.96	0.000	0.08	ns	-0.21	ns	-0.08	ns
**Malvidin-3-O-acetylGlc**	0.98	0.000	0.01	ns	-0.01	ns	0.02	ns
**Delphinidin-3-O-(6''-O-coumaroyl)Glc**	-0.77	0.000	0.14	ns	0.58	0.003	0.11	ns
**Cyanidin-3-O-(6''-O-coumaroyl)Glc**	0.99	0.000	0.00	ns	-0.11	ns	-0.04	ns
**Petunidin-3-O-(6''-O-coumaroyl)Glc**	-0.83	0.000	0.21	ns	0.45	0.028	0.06	ns
**Peonidin-3-O-(6''-O-coumaroyl)Glc**	0.92	0.000	0.14	ns	-0.30	ns	-0.10	ns
**ABA**	0.60	0.002	-0.34	ns	0.66	0.000	0.03	ns
**ABA-GE**	-0.06	ns	0.71	0.000	0.51	0.010	-0.17	ns
**DPA**	-0.70	0.000	-0.33	ns	0.17	ns	-0.27	ns
**PA**	-0.83	0.000	-0.23	ns	0.11	ns	-0.22	ns
**Caftaric acid**	0.52	0.010	0.44	0.030	-0.06	ns	0.04	ns
**Coutaric acid**	0.49	0.015	0.19	ns	0.41	0.044	0.06	ns
**Rutin and QuercetinGlc**	0.67	0.000	-0.60	0.002	0.15	ns	0.34	ns
**Myricetin**	0.41	0.046	-0.73	0.000	0.40	ns	0.24	ns
**Degradation products**	0.28	ns	-0.73	0.000	-0.53	0.007	0.03	ns

Correlation coefficients related to Figure 7B

	Axis1	*p*- value	Axis2	*p*- value	Axis3	*p*- value	Axis4	*p*- value
**Spredawn**	0.75	0.005	0.64	0.025	0.07	ns	0.08	ns
**NHH**	0.78	0.003	0.43	ns	-0.38	ns	0.10	ns
**AEBT**	0.38	ns	-0.81	0.001	0.23	ns	-0.36	ns
**Delphinidin-3-O-Glc**	-0.39	ns	0.91	0.000	-0.07	ns	0.07	ns
**Cyanidin-3-O-Glc**	0.28	ns	0.95	0.000	-0.03	ns	0.14	ns
**Petunidin-3-OGlc**	-0.57	ns	0.81	0.001	-0.06	ns	0.11	ns
**Peonidin3-O-Glc**	-0.30	ns	0.94	0.000	0.11	ns	0.13	ns
**Malvidin-3-O-Glc**	-0.91	0.000	0.39	ns	0.05	ns	0.06	ns
**Delphinidin-3-O-acetateGlc**	-0.69	0.012	0.59	0.044	0.23	ns	-0.31	ns
**Peonidin-3-O-acetylGlc**	0.60	0.038	0.46	ns	-0.48	ns	-0.07	ns
**Malvidin-3-O-acetylGlc**	-0.99	0.000	-0.09	ns	-0.01	ns	-0.09	ns
**Delphinidin-3-O-(6''-O-coumaroyl)Glc**	-0.96	0.000	-0.03	ns	0.01	ns	-0.26	ns
**Cyanidin-3-O-(6''-O-coumaroyl)Glc**	0.34	ns	0.86	0.000	-0.13	ns	-0.30	ns
**Petunidin-3-O-(6''-O-coumaroyl)Glc**	-0.98	0.000	0.03	ns	-0.13	ns	-0.16	ns
**Peonidin-3-O-(6''-O-coumaroyl)Glc**	-0.25	ns	0.67	0.017	-0.11	ns	-0.63	0.030
**Malvidin-3-O-(6''-O-coumaroyl)Glc**	-0.78	0.003	-0.03	ns	-0.45	ns	-0.33	ns
**Peonidin-3-O-(6''-O-coumaroyl)Glc + Malvidin-3-O-(6''-O-coumaroyl)Glc**	-0.99	0.000	-0.01	ns	-0.08	ns	0.00	ns
**ABA**	-0.54	ns	0.38	ns	0.53	ns	0.34	ns
**ABA-GE**	-0.84	0.001	0.23	ns	-0.30	ns	0.24	ns
**DPA**	0.37	ns	0.39	ns	0.60	0.039	-0.50	ns
**PA**	0.54	ns	0.25	ns	0.70	0.011	-0.29	ns
**Caftaric acid**	-0.72	0.008	-0.53	ns	0.23	ns	-0.35	ns
**Gallic acid deriv**	0.52	ns	0.84	0.001	0.01	ns	0.13	ns
**Coutaric acid**	-0.96	0.000	-0.06	ns	-0.03	ns	-0.19	ns
**Rutin and QuercetinGlc**	0.73	0.007	-0.29	ns	-0.36	ns	-0.42	ns
**Myricetin**	0.40	ns	0.56	ns	-0.59	0.042	-0.35	ns
**Degradation products**	0.92	0.000	0.09	ns	0.31	ns	-0.12	ns

Correlation coefficients related to Figure 7C

	Axis1	*p*- value	Axis2	*p*- value	Axis3	*p*- value	Axis4	*p*- value
**Spredawn**	0.84	0.001	0.49	ns	-0.18	ns	0.00	ns
**NHH**	0.40	ns	0.07	ns	-0.80	0.002	-0.35	ns
**AEBT**	-0.54	ns	0.84	0.001	0.08	ns	0.02	ns
**Delphinidin-3-O-Glc**	-0.95	0.000	-0.12	ns	0.03	ns	0.21	ns
**Cyanidin-3-O-Glc**	-0.97	0.000	-0.02	ns	-0.04	ns	0.07	ns
**Petunidin-3-OGlc**	-0.90	0.000	-0.40	ns	0.07	ns	0.11	ns

**Peonidin3-O-Glc**	-0.21	ns	-0.25	ns	-0.74	*0.006*	0.52	ns
**Malvidin-3-O-Glc**	-0.40	ns	-0.90	*0.000*	0.04	ns	0.09	ns
**Delphinidin-3-O-acetateGlc**	-0.91	*0.000*	0.06	ns	-0.24	ns	0.05	ns
**Malvidin-3-O-acetylGlc**	-0.20	ns	-0.86	*0.000*	-0.35	ns	-0.28	ns
**Delphinidin-3-O-(6''-O-coumaroyl)Glc**	-0.78	*0.003*	-0.18	ns	0.11	ns	-0.34	ns
**Cyanidin-3-O-(6''-O-coumaroyl)Glc**	-0.95	*0.000*	-0.03	ns	-0.02	ns	0.27	ns
**Petunidin-3-O-(6''-O-coumaroyl)Glc**	-0.87	*0.000*	-0.48	ns	0.02	ns	-0.05	ns
**Malvidin-3-O-(6''-O-coumaroyl)Glc**	-0.34	ns	-0.24	ns	-0.57	ns	-0.33	ns
**Peonidin-3-O-(6''-O-coumaroyl)Glc + Malvidin-3-O-(6''-O-coumaroyl)Glc**	0.28	ns	-0.84	*0.001*	-0.44	ns	0.05	ns
**ABA**	-0.92	*0.000*	0.29	ns	-0.04	ns	-0.20	ns
**ABA-GE**	-0.20	ns	-0.95	*0.000*	0.10	ns	-0.06	ns
**DPA**	-0.70	*0.012*	0.43	ns	0.06	ns	-0.41	ns
**PA**	-0.88	*0.000*	-0.22	ns	0.05	ns	-0.29	ns
**Caftaric acid**	0.01	ns	-0.40	ns	0.39	ns	0.32	ns
**Coutaric acid**	-0.54	ns	-0.28	ns	0.00	ns	0.21	ns
**Rutin and QuercetinGlc**	-0.56	ns	0.80	*0.002*	0.12	ns	0.14	ns
**Myricetin**	-0.61	*0.034*	0.75	*0.005*	-0.13	ns	-0.03	ns
**Degradation products**	0.27	ns	0.85	*0.000*	-0.14	ns	0.36	ns

ns : non significant
