# Supplementary material for: Grape Ripening Is Regulated by Deficit Irrigation/Elevated Temperatures According to Cluster Position in the Canopy
Source: Front Plant Sci. 2016 Nov 15;7:1640. doi: 10.3389/fpls.2016.01640 (PMC5108974; doi:10.3389/fpls.2016.01640)
Supplement: Supplementary file 4 [file Table4.PDF]

Supplementary Table 4: The Pearson's product correlation coefficient was calculated in order to disclose significant relationships between principal componentes and the variables analyzed considering ABA metabolites data set from 2014 for Tempranillo cv.

Correlation coefficients related to *véraison* (Figure 8 plot A)								
	**Axis1**	***p*- value**	**Axis2**	***p*- value**	**Axis3**	***p*- value**	**Axis4**	***p*- value**
**Spredawn**	-0.91	*0.000*	0.39	ns	0.01	ns	-0.03	ns
**NHH**	-0.65	*0.023*	0.73	*0.007*	-0.02	ns	-0.08	ns
**IET**	0.07	ns	-0.98	*0.000*	0.11	ns	-0.01	ns
**ABA**	-0.31	ns	-0.60	*0.041*	0.36	ns	0.13	ns
**ABA_GE**	0.94	*0.000*	-0.06	ns	-0.01	ns	-0.22	ns
**DPA**	-0.19	ns	-0.89	*0.000*	0.17	ns	-0.13	ns
**PA**	0.85	*0.000*	0.34	ns	-0.13	ns	0.12	ns
VviNCED1	-0.25	ns	-0.20	ns	-0.56	ns	0.74	*0.006*
VviBG1	-0.18	ns	0.21	ns	0.70	*0.011*	0.53	ns
VviHyd1	-0.55	ns	-0.55	ns	-0.45	ns	-0.09	ns
VviHyd2	-0.88	*0.000*	0.05	ns	0.06	ns	-0.40	ns
**Correlation coefficients related to mid-ripening (Figure 8 plot B)**								
	**Axis1**	***p*- value**	**Axis2**	***p*- value**	**Axis3**	***p*- value**	**Axis4**	***p*- value**
**Spredawn**	-0.97	*0.000*	-0.18	ns	0.12	ns	0.03	ns
**NHH**	-0.80	*0.002*	-0.26	ns	0.25	ns	0.20	ns
**IET**	0.49	ns	-0.65	*0.022*	0.52	ns	0.06	ns
**ABA**	0.95	*0.000*	0.09	ns	0.11	ns	0.10	ns
**ABA_GE**	0.43	ns	-0.68	*0.015*	-0.51	ns	-0.09	ns
**DPA**	0.58	*0.047*	-0.58	*0.048*	0.54	ns	0.12	ns
**PA**	0.92	*0.000*	-0.13	ns	-0.08	ns	0.14	ns
VviNCED1	0.07	ns	-0.89	*0.000*	-0.32	ns	-0.12	ns
VviBG1	-0.73	*0.008*	-0.44	ns	-0.28	ns	-0.32	ns
VviHyd1	-0.26	ns	0.02	ns	0.74	*0.005*	-0.55	ns
VviHyd2	-0.73	*0.007*	-0.29	ns	0.15	ns	0.47	ns
**Correlation coefficients related to full maturation (Figure 8 plot C)**								
	**Axis1**	***p*- value**	**Axis2**	***p*- value**	**Axis3**	***p*- value**	**Axis4**	***p*- value**
**Spredawn**	-0.88	*0.000*	-0.35	ns	-0.23	ns	0.08	ns
**NHH**	-0.45	ns	-0.02	ns	-0.77	*0.003*	-0.18	ns
**IET**	0.38	ns	-0.88	*0.000*	0.08	ns	-0.12	ns
**ABA**	0.87	*0.000*	-0.42	ns	-0.08	ns	-0.18	ns
**ABA_GE**	0.34	ns	0.89	*0.000*	0.11	ns	0.01	ns
**DPA**	0.77	*0.003*	-0.48	ns	-0.22	ns	-0.02	ns
**PA**	0.94	*0.000*	0.08	ns	-0.17	ns	-0.14	ns
VviNCED1	0.05	ns	-0.75	*0.005*	0.37	ns	0.40	ns
VviBG1	-0.76	*0.004*	0.08	ns	0.25	ns	-0.51	ns
VviHyd1	-0.41	ns	-0.45	ns	0.62	*0.031*	-0.32	ns
VviHyd2	-0.53	ns	-0.53	ns	-0.44	ns	0.08	ns

ns : non significant
