# Supplementary material for: Grape Ripening Is Regulated by Deficit Irrigation/Elevated Temperatures According to Cluster Position in the Canopy
Source: Front Plant Sci. 2016 Nov 15;7:1640. doi: 10.3389/fpls.2016.01640 (PMC5108974; doi:10.3389/fpls.2016.01640)

Supplementary Figure 1. Exemplary day curves on one day for berry temperature of exposed east cluster (Eclust), west cluster (Wclust) and average of air temperature (Avg_T_{air}). Red line corresponds to berry temperature threshold above which anthocyanin accumulation is compromised.

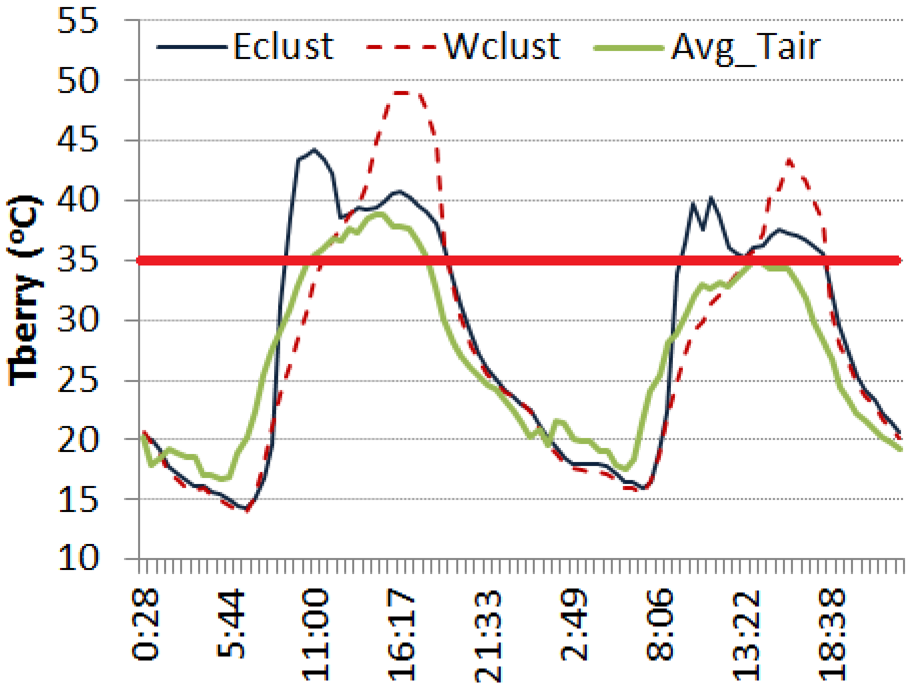

Supplement: Supplementary file 5 [file Image1.PDF]
