# Supplementary material for: Grape Ripening Is Regulated by Deficit Irrigation/Elevated Temperatures According to Cluster Position in the Canopy
Source: Front Plant Sci. 2016 Nov 15;7:1640. doi: 10.3389/fpls.2016.01640 (PMC5108974; doi:10.3389/fpls.2016.01640)
Supplement: Supplementary file 6 [file Image2.PDF]

Supplementary Figure 2. Principal components analysis performed on cumulative water stress ($S\Psi_{pd}$), NHH and AEBT* at *véraison* from 2013 (A) and 2014 (B) growing seasons for Tempranillo cv. (A) PC1=76%; PC2=16% (B) PC1=49%; PC2=35%

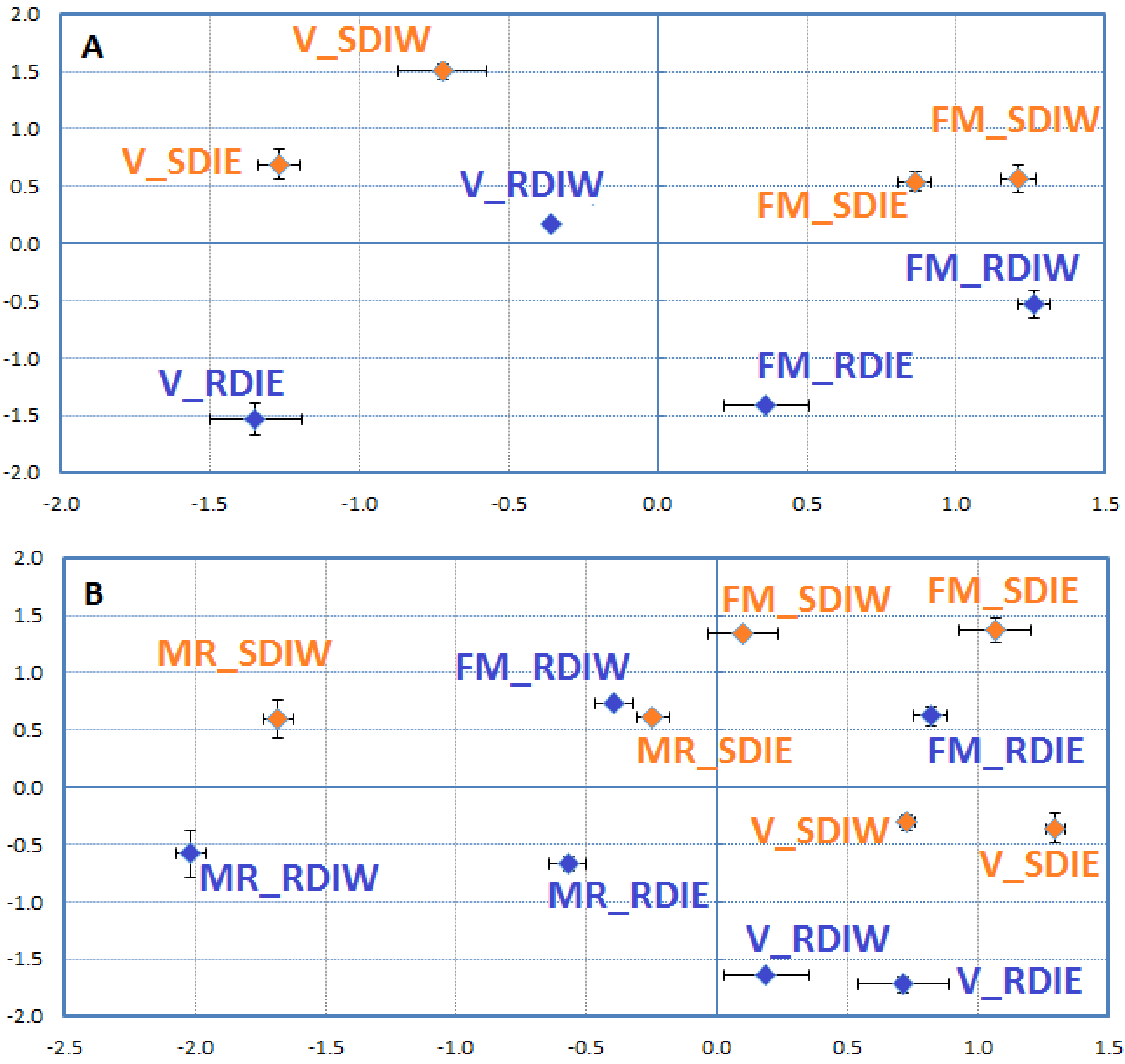

* AEBT- Accumulated Exceeding Berry Temperature was determined as follow:

$$AEBT = \begin{cases} 0, & if\ Tb \leq 35ºC \\ Tb - 35, & if\ Tb > 35ºC \end{cases}$$
